# Cholesterol depletion impairs contractile machinery in neonatal rat cardiomyocytes

**DOI:** 10.1038/srep43764

**Published:** 2017-03-03

**Authors:** Barbara Hissa, Patrick W. Oakes, Bruno Pontes, Guillermina Ramírez-San Juan, Margaret L. Gardel

**Affiliations:** 1 James Franck Institute, Institute for Biophysical Dynamics and Physics Department, University of Chicago, Chicago, IL, United States; 2 LPO-COPEA, Instituto de Ciências Biomédicas, Universidade Federal do Rio de Janeiro, Rio de Janeiro, RJ, Brazil

## Abstract

Cholesterol regulates numerous cellular processes. Depleting its synthesis in skeletal myofibers induces vacuolization and contraction impairment. However, little is known about how cholesterol reduction affects cardiomyocyte behavior. Here, we deplete cholesterol by incubating neonatal cardiomyocytes with methyl-beta-cyclodextrin. Traction force microscopy shows that lowering cholesterol increases the rate of cell contraction and generates defects in cell relaxation. Cholesterol depletion also increases membrane tension, Ca^2+^ spikes frequency and intracellular Ca^2+^ concentration. These changes can be correlated with modifications in caveolin-3 and L-Type Ca^2+^ channel distributions across the sarcolemma. Channel regulation is also compromised since cAMP-dependent PKA activity is enhanced, increasing the probability of L-Type Ca^2+^ channel opening events. Immunofluorescence reveals that cholesterol depletion abrogates sarcomeric organization, changing spacing and alignment of α-actinin bands due to increase in proteolytic activity of calpain. We propose a mechanism in which cholesterol depletion triggers a signaling cascade, culminating with contraction impairment and myofibril disruption in cardiomyocytes.

Cholesterol plays a fundamental role in regulating plasma membrane fluidity, integrity, and in compartmentalizing intracellular signaling events^1^. In non-muscle cells, such as fibroblasts and lymphoblasts, cholesterol depletion reorganizes the actin cytoskeleton architecture and reduces lateral mobility of membrane-bound proteins in a phosphatidylinositol 4,5- biphosphate dependent way^2^. Cholesterol-depleted endothelial cells^3^ and fibroblasts^4^ also show an increase in cell stiffness due to actin remodeling and that is probably due to an enhancement in membrane-cytoskeleton adhesion energy^5^. In skeletal muscle cells, cholesterol depletion impairs contraction, causes vacuolization and morphological abnormalities and uncouples a transmembrane glycoprotein, β-dystroglycan that connects the extracellular matrix to the cytoskeleton of those cells^6^,^7^,^8^. However, less is known about how cholesterol depletion might affect cardiomyocyte physiology and contraction dynamics.

Flask-shaped and cholesterol enriched domains, known as caveolae, are fundamental for cardiomyocyte functioning^9^. Caveolins are proteins that reside in caveolae and caveolin-3 is the most abundant muscle isoform^10^,^11^. These proteins are also important for mechanosensing^12^,^13^ and membrane tension regulation^14^ in muscle cells. Aside from modulating cellular mechanics, caveolae also regulate Ca^2+^ dynamics in cardiomyocytes being responsible for gathering and regulating the activity of important ion channels, such as L-Type Ca^2+^ channel (LTCC)^15^. Besides, caveolae localization guarantees proper β1 and β2-adrenergic receptors (β-ARs) signaling in cardiomyocytes^16^,^17^,^18^ that play a major role in the initiation of Ca^2+^ cycling in those cells.

Extracellular Ca^2+^ entry is an important step for Ca^2+^ cycling within the cardiomyocyte and it is mediated by LTCC. Regulation of LTCC opening and closing events is fundamental to guarantee a synchronous Ca^2+^ signaling^19^ that ultimately leads to cardiomyocyte contraction. β1 and β2-adrenergic receptors (β-ARs) are G-protein coupled receptors that initiate the cascade of events that culminate with the opening of a LTCC. Under agonist stimulation, β-ARs are activated and trigger cAMP production by adenylyl cyclase. cAMP activates protein kinase A (PKA) which in turn phosphorylates Ca_v_1.2, the pore-forming subunit of LTCC, keeping it open and promoting extracellular Ca^2+^ entry to the sarcoplasm^20^. It is known that extracellular Ca^2+^ plays a pivotal role in neonatal cardiomyocyte contraction since those cells lack t-tubules^11^ that are fundamental structures for the Ca^2+^-induced-Ca^2+^-release mechanism for contraction in adult cardiomyocytes^21^. Besides, neonatal cardiomyocytes also show a more pronounced increase in β2-AR-dependent cAMP concentration that activates PKA in those cells^22^.

Not only Ca^2+^ but also myofibrillar organization and stability are critical for muscle cell contraction. Myocytes have an extremely organized and stable actomyosin network that is responsible for uniform and long-range contraction^23^. This actomyosin cytoskeleton has a striated pattern that arises from sarcomere sequential positioning being each sarcomere the contractile unit of the myofibril^24^. Each sarcomere is delimited by two z-lines where actin barbed ends localize. The z-lines also contain α-actinin that is fundamental for organizing the myofibrils during development^25^. The contraction of the myofibers happen when Ca^2+^ binds to troponin-C changing tropomyosin conformation and releasing the site where myosin binds to actin allowing the filaments to slide past each other^25^. Buffering of Ca^2+^ and release of this ion to either the sarcoplasmic reticulum or to the extracellular milieu is important for proper relaxation of the myofibers since Ca^2+^ mishandling can cause cardiac issues^26^. Moreover, excess in sarcoplasmic Ca^2+^ can trigger activation of proteases like calpains^27^ that can degrade sarcomeric proteins and abrogate myofibrillar organization and contractility^28^.

To investigate the importance of cholesterol in regulating cardiomyocyte physiology we incubated the cells with a cyclodextrin called methyl-beta cyclodextrin (MβCD) to acutely deplete plasma membrane cholesterol in isolated primary neonatal rat cardiomyocytes. MβCD is a cyclic oligosaccharide that has a hydrophobic core (with a high affinity for cholesterol molecules) and a hydrophilic surface that facilitates its solubilization in aqueous media^29^ and does not have the pleiotropic effects that other cholesterol lowering drugs have, such as depletion of isoprenoids^30^. MβCD takes advantage of the cholesterol efflux dynamics in order to trap the molecule inside its cage: once the cyclodextrin gets closer to the plasma membrane, cholesterol migrates from the membrane to the MβCD core without having to be in direct contact with the aqueous media^31^. Cyclodextrins similar to MβCD can be used *in vivo* as a way of facilitating the reverse cholesterol transport in which accumulated cholesterol is removed from the vessel walls and transported to the liver for degradation decreasing the incidence of atherosclerosis in hyperlipidemic rabbits^32^.

Traction Force Microscopy (TFM) experiments revealed that cells with lower cholesterol levels had higher and variable contraction rates and relaxation defects. MβCD treatment increased the cardiomyocytes cortical mechanical properties values, the frequency of Ca^2+^ spikes and intracellular Ca^2+^ concentration. We correlated these results with the fact that caveolin-3 and Ca_v_1.2 change distribution across the sarcolemma upon cholesterol depletion which, in turn, might impact the regulation of the LTCC. In order to test this hypothesis we measured cAMP-dependent PKA activation and we saw an increase in the enzymatic activity consequent to cholesterol depletion. This result corroborates the increase in intracellular Ca^2+^ after MβCD treatment since PKA activity is directly involved in Ca_v_1.2 opening events. Immunofluorescence revealed that cholesterol depletion abrogated the organization of myofibrils, changing the spacing and alignment of α-actinin bands and this change was correlated with increase in proteolytic activity of calpain probably due to enhancement in cytoplasmic Ca^2+^ concentration.

This work suggests for the first time a complete mechanism by which cholesterol depletion, due to MβCD incubation, impairs contraction in neonatal cardiomyocytes.

## Materials and Methods

For more details in the methods section visit the supplemental information that is available online.

### Ethics statement

All animals were maintained at The University of Chicago Animal Resources Center. Animal husbandry and all experimental procedures were performed in compliance with the guidelines of the Institutional Animal Care and Use Committee (IACUC). Experimental protocols were approved by the University of Chicago IACUC (protocol number 72347).

### Primary culture of neonatal rat cardiomyocytes

Neonatal rat hearts (p0-p3) were submitted to enzymatic digestion steps that were adapted from a previously stablished protocol^33^. Purified cardiomyocytes were collected, plated either on round glass coverslips coated with fibronectin alone or containing fibronectin crosslinked to polyacrylamide (PAA) gels.

### Drug treatments

To deplete cholesterol the cardiomyocytes were incubated with either 5.0 or 7.5 mmol/L MβCD, for 45 minutes, at 37 °C.

### Immunofluorescence

After fixation, cardiomyocytes were incubated with the following primary antibodies: mouse monoclonal anti-sarcomeric α-actinin, rabbit polyclonal anti-caveolin-3, mouse monoclonal Ca_v_1.2. Next, the cells were rinsed and incubated with secondary fluorescent antibodies. Images of cells were taken on Ti-E Nikon inverted confocal microscope using either 60 × 1.49 NA ApoTIRF or 40 × 1.30 Plan Fluor oil-immersion objectives.

### Traction Force Microscopy

Cardiomyocytes were plated on polyacrylamide gels with a shear modulus of 8.64 KPa embedded with fluorescent beads as previously described^34^,^35^,^36^. Images of the fluorescent beads with and without the cells on top were acquired and analyzed using MATLAB scripts previously developed^34^,^35^,^37^,^38^,^39^,^40^.

### Tether extraction with optical tweezers

An infrared optical tweezers (OT) setup was used to extract tethers from the surface of the cardiomyocytes. The OT setup and force calibration were performed as previously described^41^,^42^. Scanning Electron Microscopy (SEM) was used to measure the tether radii^4^,^41^,^42^.

### Imaging of calcium sparks

Epifluorescence signal from the Fluo-4 AM Ca^2+^ probe (Thermo Fisher Scientific) was acquired for control and treated cardiomyocytes and cytoplasmic Ca^2+^ sparks were analyzed using ImageJ. Conversion of fluorescence values into Ca^2+^ concentration was based on the following equation^43^:


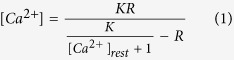


K is the dissociation constant of the Ca^2+^ dye used, R is the fluorescence ratio 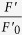 (F′_0_ is the minimum fluorescence recorder for a specific cell analyzed), [Ca^2+^]_rest_ is approximately 140 nM^44^.

### Measurement of PKA and calpain enzymatic activities

To measure cAMP-mediated PKA or calpain activities, cell extracts were submitted to either enzymatic assay using PKA activity kit (Enzo Life Sciences) or using calpain Activity Fluorometric Assay Kit (BioVision Inc) according to manufacturer’s instructions.

### Statistical analysis and data quantification

Student’s t-test was used to compare between control and treated groups. Data was represented by mean values ± standard error unless otherwise stated. The experimental errors for F_0_ and R were propagated by taking the derivatives of the equations 2 and 3 with respect to both F_0_ and R. Statistical differences were labeled with asterisks. Fourier analysis of contraction and Ca^2+^ sparks as well as quantification of α-actinin bands were done using MATLAB scripts described in the supplemental material.

## Results

### Cholesterol depletion impairs contraction in rat neonatal cardiomyocytes

**Figure 1:**
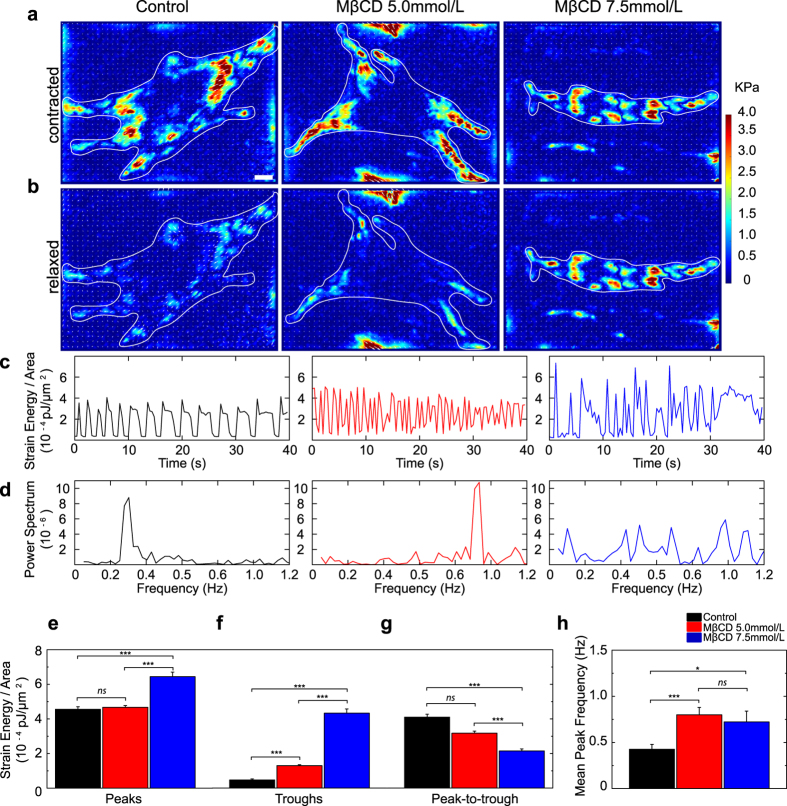
Cholesterol depletion impairs cardiomyocyte contractility. Heat maps depicting magnitudes of traction stresses of control, MβCD 5.0 and 7.5 mmol/L treated cells when contracting (**a**) and relaxing (**b**). Scale bar 10 μm. White contours indicate cell outlines, white arrows indicate direction of traction forces on PAA substrates and the heat scale (in KPa) is indicated on the right. (**c**) Line plots showing variations of strain energy per cell area for control, MβCD 5.0 and 7.5 mmol/L treated cardiomyocytes. (**d**) Power spectrum showing the characteristic frequencies of strain energy per area. Mean and standard error for strain energy per cell area peaks (**e**), troughs (**f**), peak-to-trough distances (**g**) and peak frequencies (**h**) for control (n = 17), MβCD 5.0 (n = 21) and MβCD 7.5 mmol/L (n = 15). ***p < 0.0001, *p < 0.05, ns = not statistically different.

To deplete plasma membrane cholesterol we used a cyclodextrin called MβCD. To directly measure how cholesterol depletion affects contractility of neonatal cardiomyocytes, we used time-resolved Traction Force Microscopy (TFM). Cell contractility is defined as the ability of the cell to generate contractile forces via actomyosin interactions within myofibrils. For the case of cells attached to a flat substrate, we can measure their contractile behavior using TFM. Traction stress maps were obtained for 40 seconds to capture the spontaneous beating of cardiomyocytes plated on fibronectin coated gels. The maximal and minimal traction stresses obtained during this time lapse are shown in Fig. 1a and b, respectively, and correspond to the maximally contracted and relaxed states of the cardiomyocyte. The strain energy is a measure of the total mechanical work done by the cell in deforming the substrate. The ratio of strain energy to cell area defines a characteristic contractility measurement that is independent of cell shape and size^39^. The quantification of the strain energy per cell area from the traction stress maps demonstrates that control cardiomyocytes exhibit periodic contraction-relaxation patterns (Fig. 1c), with a majority of the power exerted at a frequency of 0.3 Hz (Fig. 1d). The contraction becomes more irregular in cholesterol depleted cells (Fig. 1a–d). Treatment with 7.5 mmol/L MβCD increased both the energy of the peaks (Fig. 1e) and the troughs (Fig. 1f) which suggests impairment in cell relaxation. In order to evaluate the resultant strain energy per cell area we plotted the difference between peak and trough strain energy/cell area (Fig. 1g) and we found that the overall contractility decreases. Moreover, the power spectra show that cholesterol depletion yielded noisier spectra (Fig. 1d) and increased the beating frequency (Fig. 1h). Thus, cholesterol depletion severely affects neonatal cardiomyocytes contractility.

### Cholesterol depletion increases cortical mechanical properties values of neonatal rat cardiomyocytes

**Figure 2:**
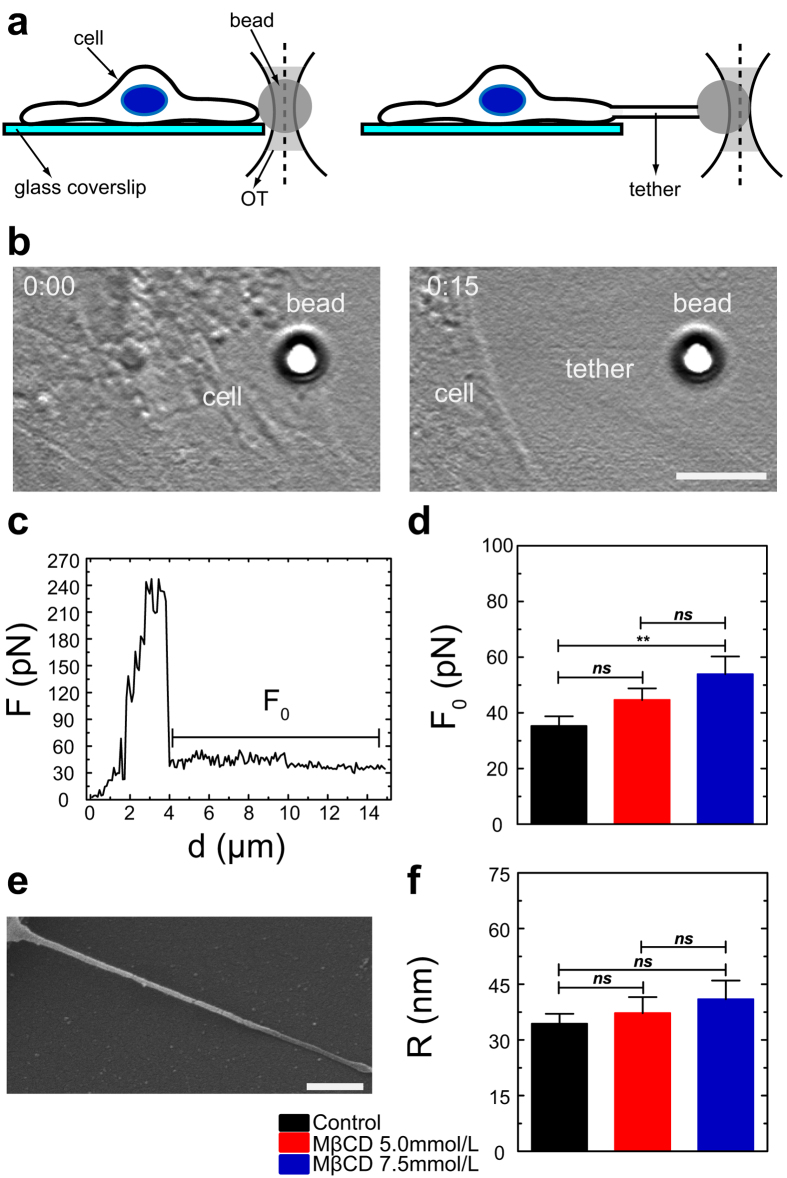
Cholesterol depletion changes cortical mechanical properties of cardiomyocytes. (**a**) Cartoon of a cardiomyocyte and an optically-trapped polystyrene bead attached to its surface (left) and formation of the tether tube (right). (**b**) Bright field image of a real cardiomyocyte represented in (**a**) before (0:00 s, left) and after (0:15 s, right) tether extraction. Scale bar 5 μm. (**c**) Typical Force *F* versus displacement *d* of the bead center of mass during a typical tether extraction experiment for a control cardiomyocyte. (**d**) Mean and standard error for *F*_*0*_ values of control (n = 40) and cholesterol depleted cardiomyocytes treated with either 5.0 (n = 40) or 7.5 mmol/L MβCD (n = 20). (**e**) Representative SEM image of a tether extracted from a control cardiomyocyte. Scale bar 500 nm. (**f**) Mean and standard error for tether radii of control (n = 6), MβCD 5.0 (n = 7) and 7.5 mmol/L (n = 3) treated cardiomyocytes. **p < 0.01, ns = not statistically different.

To determine how cholesterol depletion altered cortical mechanical properties of neonatal rat cardiomyocytes, tethers were extracted from the surfaces of these cells using OT. In this assay, OT were used to trap and control the position of an uncoated polystyrene bead that forms a tight bond with the cell surface when it is in close proximity (Fig. 2a and b, left). As the position of the bead and the cell are varied, a tether is formed (Fig. 2a and b, right). By using OT calibration, it is possible to obtain the force as a function of distance between the cell and bead (Fig. 2c). When the bead starts to be pulled away from the cell surface, the applied force increases up to a maximum value (F_m_) and then abruptly decreases to a constant value F_0_. F_m_ corresponds to the moment that precedes the tether formation whereas F_0_ corresponds to the force applied to form and elongate the tether. We measured F_0_ for all tested conditions (Fig. 2d) and saw a trend in increasing this value for 5.0 mmol/L MβCD treated cells (45 ± 4 pN) and a statistical increase for cells treated with 7.5 mmol/L MβCD (54 ± 6 pN) in comparison to control cardiomyocytes (35 ± 4 pN).

Tethers radii were also measured by SEM (Fig. 2e). Comparisons between radii values indicated no statistical differences between control and cholesterol depleted groups (Fig. 2f).

In order to calculate the membrane tension σ and bending rigidity κ we used equations (2) and (3):


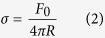



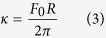


by substituting the values for F_0_ and R in the equations (2) and (3) we obtained σ and κ for the tested groups (Table 1).

Based on those results it is possible to affirm that cholesterol depletion increases both cortical mechanical properties values (σ and κ).

In order to uncouple contributions from the plasma membrane and from the cytoskeleton to cortical mechanical properties we decided to induce plasma membrane vesicles (PMVs) (depicted in Fig. S1A) in the cardiomyocytes (for technical information, please see supplementary information section). By doing that, we were able to measure specifically the contribution of the plasma membrane since the PMVs are devoid of cytoskeletal components. In order to calculate σ and κ for the tethers extracted from PMVs it is necessary to obtain the tethers’ radii. However, since PMVs are fragile and cannot resist to the fixation process and sample preparation for SEM, we used equation (4) to calculate the PMVs tethers’ radii^45^:


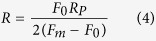


where F_m_ is the force to extract the tether from the PMV, F_0_ is the force to elongate the tether and R_p_ is the patch radius, defined in Fig. S1D. Mean values and their respective standard errors for F_m_ and F_0_ are shown in Figs S1B and S1C. PMVs’ membrane tension and bending modulus, amongst other values, are listed on Table S1.

Values for PMVs’ membrane tension are higher when cholesterol is depleted whereas there are no differences in bending moduli values between control and treated groups. Thus, contribution for enhancement in bending rigidity in cardiomyocytes comes almost exclusively from the cytoskeleton component.

### Cholesterol depletion disorganizes neonatal cardiomyocytes myofibers

To investigate whether altered contractile properties coincided with changes in the myofibril architecture, cells were fixed and stained for actin and sarcomeric α-actinin. Figure S2, left panel, shows actin cytoskeleton of control (Fig. S2A), 5.0 mmol/L (Fig. S2B) and 7.5 mmol/L (Fig. S2C) MβCD treated cardiomyocytes. Cholesterol depletion impacts significantly the organization of thin filaments within the myofibers. α-actinin labeling shows that lack of cholesterol also makes the z-bands distorted in comparison to control cells which have equally spaced and parallel z-bands (Fig. S2A).

**Figure 3:**
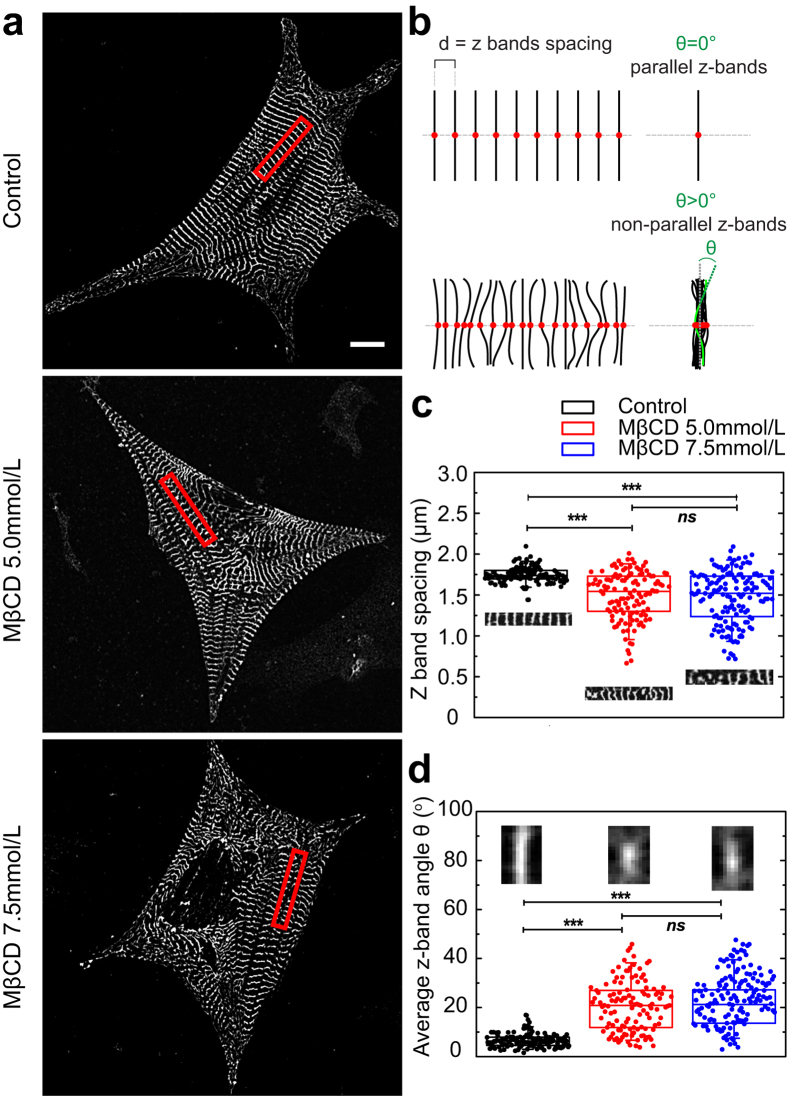
Cholesterol depletion disorganizes z-bands of primary neonatal cardiomyocytes. (**a**) Images of cardiac z-bands labelled with sarcomeric α-actinin antibody. Upper panel: control, mid panel: MβCD 5.0 and lower panel MβCD: 7.5 mmol/L. Red rectangles show approximated regions where myofibers were selected for analysis. (**b**) Cartoon depicting how the distances d and angles θ between z-bands were calculated. Upper panel shows an ideal myofiber with parallel and aligned z-bands whereas the bottom panel shows a more realistic myofiber from the cholesterol depleted case where the separation and angle between the z-bands are not uniform. Note that the centers of each myofiber are superimposed (right side) and the angle θ is defined for a particular z-band in green. (**c**) Quantification of α-actinin average spacing d between bands. Insets show regions indicated by red rectangles in A. (**d**) Quantification of mean angles θ between α-actinin bands. Insets show superposed α-actinin bands from selected myofibrils in A. Box-and-wisker plots and data spread points of control (n = 30), MβCD 5.0 (n = 30) and 7.5 mmol/L (n = 32) treated cardiomyocytes. The box-and-wisker plots indicate median (middle line), 25 and 75% of the values (smaller and bigger rectangles, respectively), 5 and 95% values are represented by the upper and lower wiskers respectively. **p < 0.001, ***p < 0.0001, ns = not statistically different according to Student’s t-test. Black boxplots and points represent control group, red boxplots and points represent MβCD 5 mmol/L treated cardiomyocytes and blue boxplots and points represent MβCD 7.5 mmol/L treated cardiomyocytes. Scale bar 10 μm.

To quantify changes in myofibrillar α-actinin organization, image analyses were performed to measure the separation distance and angle between z-bands. In control cardiomyocytes, the spacing between z-bands is quite regular, with a mean value of (1.75 ± 0.09) μm (Fig. 3a,c). In contrast, cholesterol depleted cardiomyocytes show a reduction in mean band spacing ((1.49 ± 0.29) and (1.47 ± 0.31) μm for 5.0 and 7.5 mmol/L MβCD treated cells respectively) and with a much larger distribution around the mean value for both conditions (Fig. 3c). Figure 3d shows that control cardiomyocytes have more parallel aligned z-bands within a myofibril and the angle between them is small (mean value (6.8 ± 2.3)°), as expected. As an illustrative output of the MATLAB script, the superposition of neighboring z-bands within a myofibril (Fig. 3d, inset on the left) also shows a well-defined line that represents the degree of band alignment for the control group (Fig. 3b shows a cartoon depicting how the distances and angles between z-bands were calculated). In contrast, cholesterol depleted cardiomyocytes have less aligned z-bands and that is shown in Fig. 3a (mid and bottom panels). Illustrative outputs show a broader and smeared intensity distribution for the superposition of the z-bands and that is quantified in the graph (Fig. 3d) ((20 ± 10)° for the 5.0 mmol/L MβCD case and (22 ± 10)° for the 7.5 mmol/L MβCD case) (insets in the middle and on the right show superposition of z-bands from selected myofibers, in Fig. 3a).

### Cholesterol depletion changes caveolin-3 distribution on cardiomyocytes

**Figure 4:**
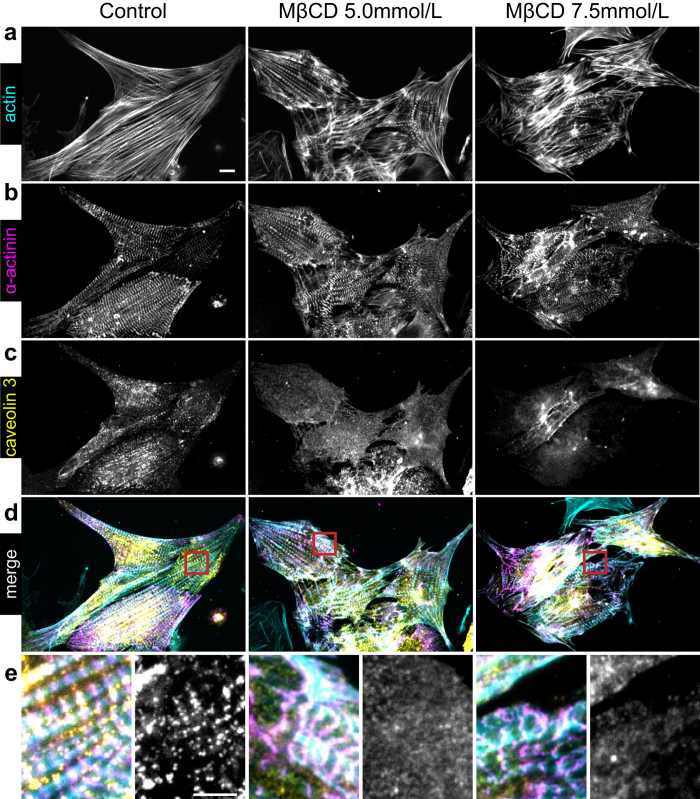
Cholesterol depletion changes caveolin-3 distribution on cardiomyocytes. (**a**) Phalloidin staining showing actin for control, 5.0 and 7.5 mmol/L MβCD treated cardiomyocytes. Scale bar 10 μm. Immunostaining of α-actinin (**b**), caveolin-3 (**c**) and superposition of the previous channels (**d**). Red rectangles in D show zoomed regions in (**e**) representing the merge and caveolin-3 channel respectively. Scale bar 5 μm.

Caveolin-3, localized in cholesterol invaginated domains known as caveolae, has been previously shown to be important for adult cardiomyocytes to adapt to sudden changes in membrane tension^14^. Moreover, actin cytoskeleton also modulates caveolin organization^46^,^47^. Since cholesterol depletion changes both membrane tension and actin cytoskeleton architecture, we decided to investigate whether caveolin-3 distribution was affected. Cardiomyocytes were fixed and labeled for actin (Fig. 4a), α-actinin (Fig. 4b) and caveolin-3 (Fig. 4c), a caveolae-specific scaffolding protein highly expressed in muscle cells^10^. Control cardiomyocytes show a punctate distribution of caveolin-3 that is mostly localized along the z-bands (Fig. 4c,d and e, left panels). After cholesterol depletion with either 5.0 (Fig. 4c,d and e, middle panels) or 7.5 mmol/L MβCD (Fig. 4c,d and e, right panels), caveolin-3 distribution becomes more homogeneous and those punctate domains, that appear in control cells, disappear. Quantification of caveolin-3 redistribution is shown in Fig. S3. Cholesterol depletion disorganizes not only cytoskeleton architecture but also caveolin-3 distribution along the z-bands.

### Cholesterol depletion changes cytoplasmic calcium spikes dynamics in cardiomyocytes

**Figure 5:**
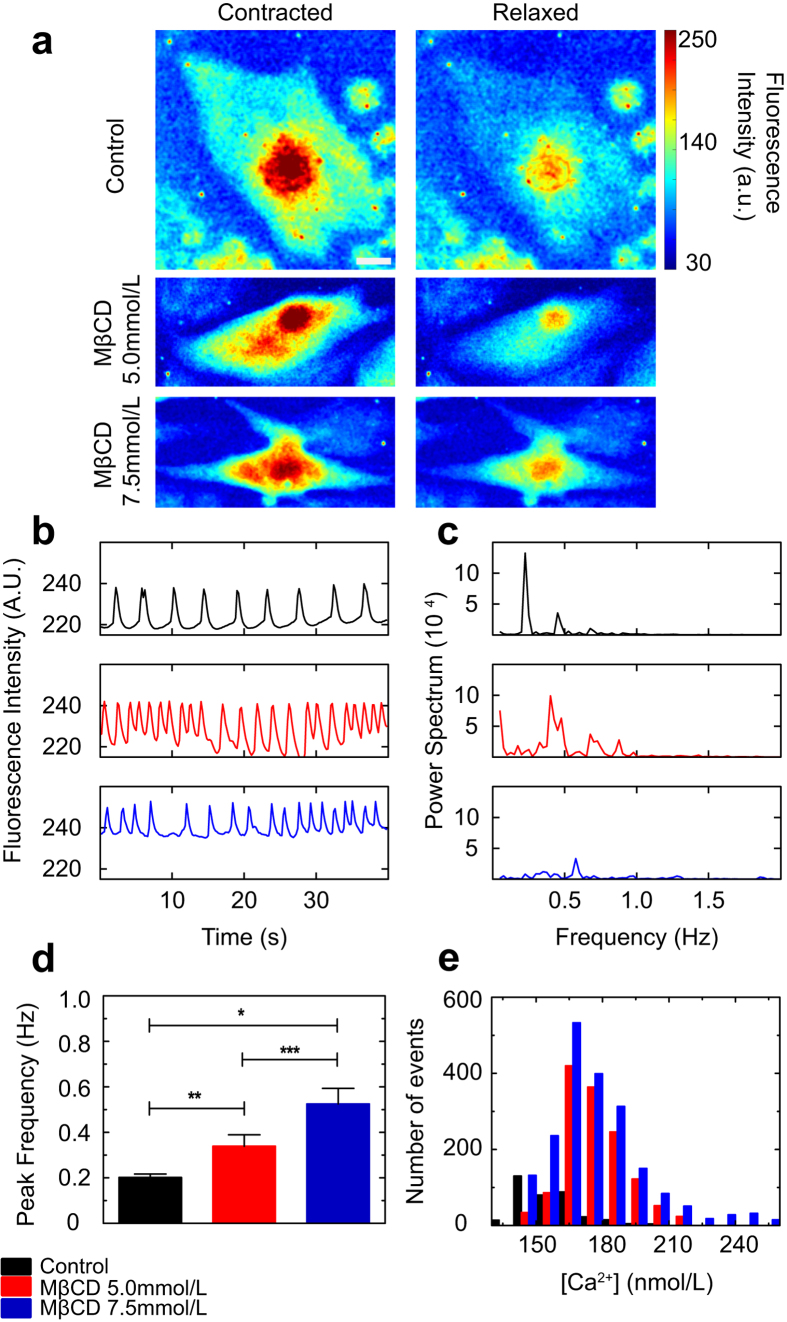
Cholesterol depletion changes calcium sparks dynamics in cardiomyocytes. (**a**) Fluorescence heat map showing Fluo-4 signal in control and cholesterol depleted cardiomyocytes during maximum contraction (panels on the left) and relaxation (panels on the right). Heat scale (arbitrary units) on the right. (**b**) Fluorescence intensity variation during 40 seconds of live cell experiment for control (top panel), MβCD 5.0 (mid panel) and 7.5 mmol/L (lower panel) treated cardiomyocytes. (**c**) Power spectrum showing characteristic frequencies of Ca^2+^ sparks for control (top panel), MβCD 5.0 (mid panel) and 7.5 mmol/L (lower panel) treated cardiomyocytes. (**d**) Mean and standard error for calcium sparks peak frequency for control (n = 50), MβCD 5.0 (n = 50) and MβCD 7.5 mmol/L (n = 50). ***p < 0.0001, **p < 0.01, *p < 0.05, ns = not statistically different. (**e**) Histogram showing Ca^2+^ concentration values (in nmol/L) for control (n = 50), MβCD 5.0 (n = 65) and MβCD 7.5 mmol/L (n = 72).

So far, we demonstrated that cholesterol depletion impaired actin cytoskeleton organization and contractility of the cardiomyocytes. We also showed that cholesterol depletion changed the cortical mechanical properties as well as caveolin-3 distribution in cardiomyocytes. Since Ca^2+^ is another fundamental piece of the contractile machinery in muscle cells, we sought to investigate whether cholesterol depletion was also changing sarcoplasmic Ca^2+^ cycling in cardiomyocytes. For that, control and cholesterol depleted cardiomyocytes were incubated with Fluo-4 AM and imaged in order to track changes in intracellular calcium spike dynamics. In Fig. 5a the heat maps show Ca^2+^ fluorescence for control and MβCD treated cardiomyocytes in the contracted and relaxed states. By comparing contracted states, we see that cytoplasmic Ca^2+^ fluorescence intensity increases when cholesterol is depleted. For the relaxed states we also noticed that cholesterol depleted cells cannot buffer the cytoplasmic Ca^2+^ efficiently during relaxation. Quantification of intracellular Ca^2+^ fluorescence (Fig. 5b) reveals that the oscillation in Ca^2+^ spikes is periodic in the control case, and during the relaxation period the fluorescence signal drops almost to zero. On the other hand, cardiomyocytes with lower cholesterol levels, especially the cells treated with 7.5 mmol/L MβCD, have a higher baseline for calcium intensity and during relaxation the calcium is not buffered as efficiently as it is in control cells. Moreover, we observe noisier spectra for calcium spikes in cholesterol-depleted cells and this feature is quantified in the power spectra graphs (Fig. 5c). We not only see an increase in contraction rate (Fig. 1) but we also observe an increase in calcium peak frequency as cholesterol is depleted from the cardiomyocytes (Fig. 5d). By quantifying the cytoplasmic Ca^2+^ concentration (see equation 1) we confirmed that cholesterol depleted cardiomyocytes presented more events where Ca^2+^ concentration was higher in comparison to control cells (Fig. 5e). In accordance with TFM results, the Ca^2+^ spike data show that cholesterol depletion changes cytoplasmic Ca^2+^ dynamics leading to enhancement in both Ca^2+^ concentration and frequency of Ca^2+^ spikes and impairment in cell buffering of cytoplasmic Ca^2+^ when the cells undergo relaxation.

### Cholesterol depletion impairs L-type Ca^2+^ channel localization at plasma membrane of cardiomyocytes triggering PKA and calpain activation

**Figure 6:**
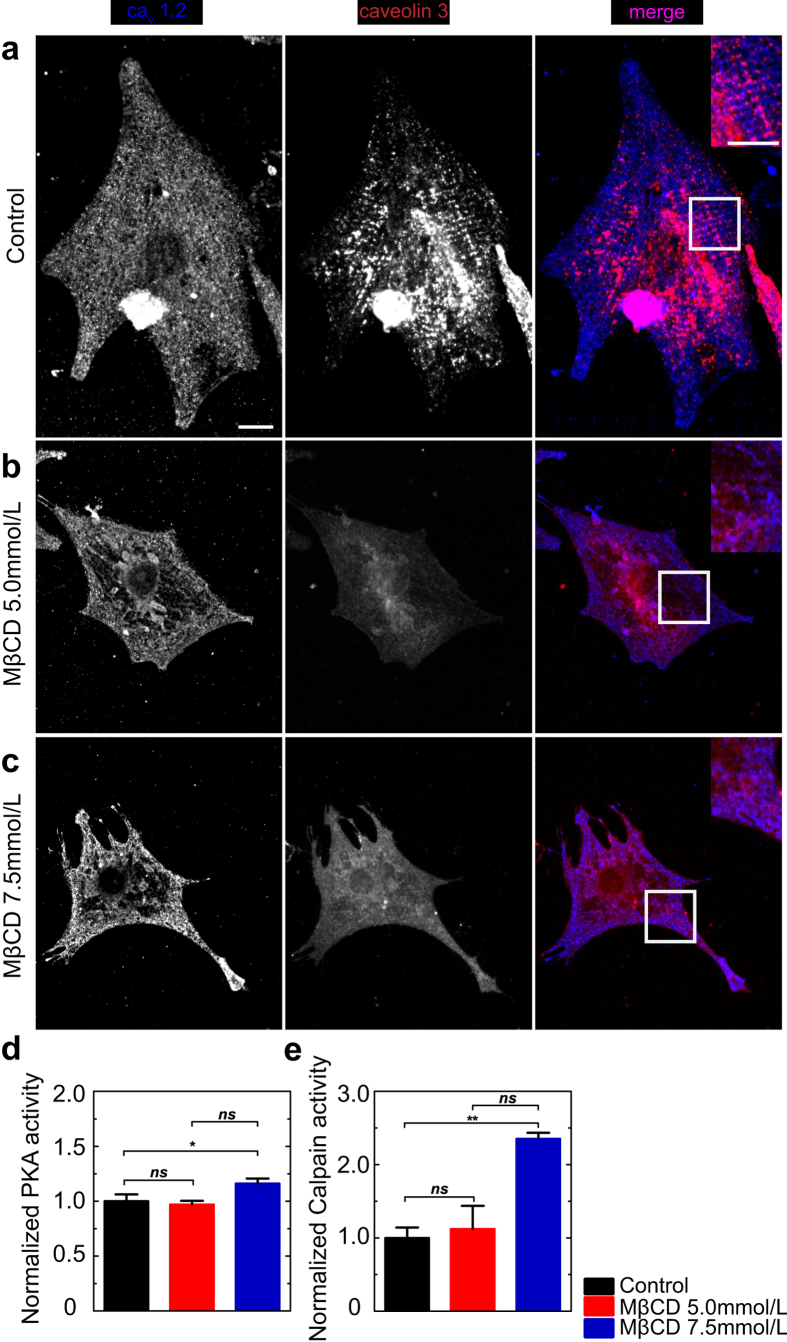
Cholesterol depletion changes distribution of LTCC and activates PKA on cardiomyocytes. Immunostaining for subunit Ca_v_1.2 of LTCC, caveolin-3 and superposition of the two channels from control (**a**), 5.0 (**b**) and 7.5 mmol/L MβCD (**c**) treated cardiomyocytes. White squares on the merged images represent regions that were zoomed on the insets. Scale bar 10 μm. (**d**) Absorbance measurements indicating normalized enzymatic activity of PKA and (**e**) normalized values for calpain activity measured from cell extracts of control, MβCD 5.0 mmol/L and MβCD 7.5 mmol/L treated cardiomyocytes. PKA and calpain enzymatic assays were performed using triplicates per each condition **p < 0.01, *p < 0.05, ns = not statistically different according to Student’s t-test.

Since extracellular Ca^2+^ is critical for neonatal cardiomyocyte contraction^44^,^48^,^49^,^50^,^51^, the interaction between the sarcolemma and extracellular milieu is crucial to guarantee homeostasis during contraction. We saw that cholesterol depletion increases membrane tension and disrupts caveolin-3 distribution. Since caveolar domains contain Ca^2+^ channels, such as LTCC, and regulate their activity^52^, we decided to investigate whether cholesterol depletion was also changing localization of LTCC in the sarcolemma of neonatal cardiomyocytes. To do that, cardiomyocytes were fixed and labeled for LTCC subunit Ca_v_1.2 (also known as α_1c_ pore-forming subunit, highly expressed in cardiac muscle^53^) (Fig. 6, left pannel) and caveolin-3 (Fig. 6, mid panel). Control cardiomyocytes (Fig. 6a) exhibit a homogenous distribution of the Ca_v_1.2 labeling (Fig. 6a, left panel, inset). However, after cholesterol depletion with either 5.0 (Fig. 6b) or 7.5 mmol/L (Fig. 6c) MβCD the distribution of the Ca_v_1.2 subunit becomes less uniform with areas of the cells that are devoid of labeling, especially at perinuclear regions (compare Fig. 6a,b and c, left panels. See Fig. S4 for quantification of Ca_v_1.2 distribution). It is known that PKA phosphorylates Ca_v_1.2 in order to control the opening of the LTCC pore^54^. In order to correlate changes in localization of LTCC with its function we performed an enzymatic assay to measure cAMP-dependent PKA activity in control and cholesterol depleted cardiomyocytes. Figure 6d shows that 7.5 mmol/L MβCD elicits enhancement in cAMP-dependent PKA activity in cardiomyocytes in comparison to control cells leading to more pore-opening events, which allow Ca^2+^ entry. In conjunction with our previous results, this evidence suggests that cholesterol depletion activates cAMP-dependent PKA activity leading to enhancement in Ca^2+^ entry, which in turn deregulates contraction dynamics in neonatal cardiomyocytes. In addition, the clear disruption of myofibers in cells treated with MβCD raised the question whether some Ca^2+^ -dependent cytoplasmic protease might have been activated in the process. Since we saw an increase in cytoplasmic Ca^2+^ concentration we decided to test activity of calpain, one of the main proteases in muscle tissue that is activated by intracellular Ca^2+^ 27. Normalized values for calpain activity showed that MβCD treatment increased calpain activity in neonatal cardiomyocytes in comparison to control cells. This result corroborates the idea that this Ca^2+^-dependent protease might be degrading the myofibers due to mishandling of cytoplasmic Ca^2+^ caused by cholesterol depletion.

## Discussion

It is widely known that cholesterol plays a pivotal role in controlling permeability, diffusion and receptor clustering at the plasma membrane level^55^. In non-muscle cells, cholesterol regulates actin cytoskeleton architecture and cortical mechanical properties^2^,^3^,^4^,^5^ whereas in smooth and skeletal muscle cells, cholesterol is important for modulating the contraction of these cells^6^,^7^,^8^,^56^. However, less is known about how cholesterol affects cardiomyocyte physiology and how its depletion affects cell contractility.

The contractility of cardiomyocytes is controlled by two different actin cytoskeletal architectures: sarcomeric myofibrils and the sub-sarcolemmal actin. There is substantial evidence that the sub-sarcolemmal actin contractility is regulated in a similar manner as in non-muscle cells^57^. By using MβCD incubation, an acute and very specific method for depleting cholesterol from the plasma membrane^31^,^58^, this work showed important features acquired by neonatal ventricular rat cardiomyocytes when submitted to cholesterol depletion. As expected, similarly to what was previously observed for immortalized fibroblasts^4^, OT-mediated tether extraction, revealed an increase in both membrane tension and bending rigidity of the cardiomyocytes submitted to MβCD incubation. Tether extraction on PMVs also showed an increase in membrane tension but not in bending rigidity pointing out to an exclusive contribution from the sub-sarcolemmal actin cytoskeleton to bending rigidity increase in cholesterol-depleted cardiomyocytes.

**Figure 7:**
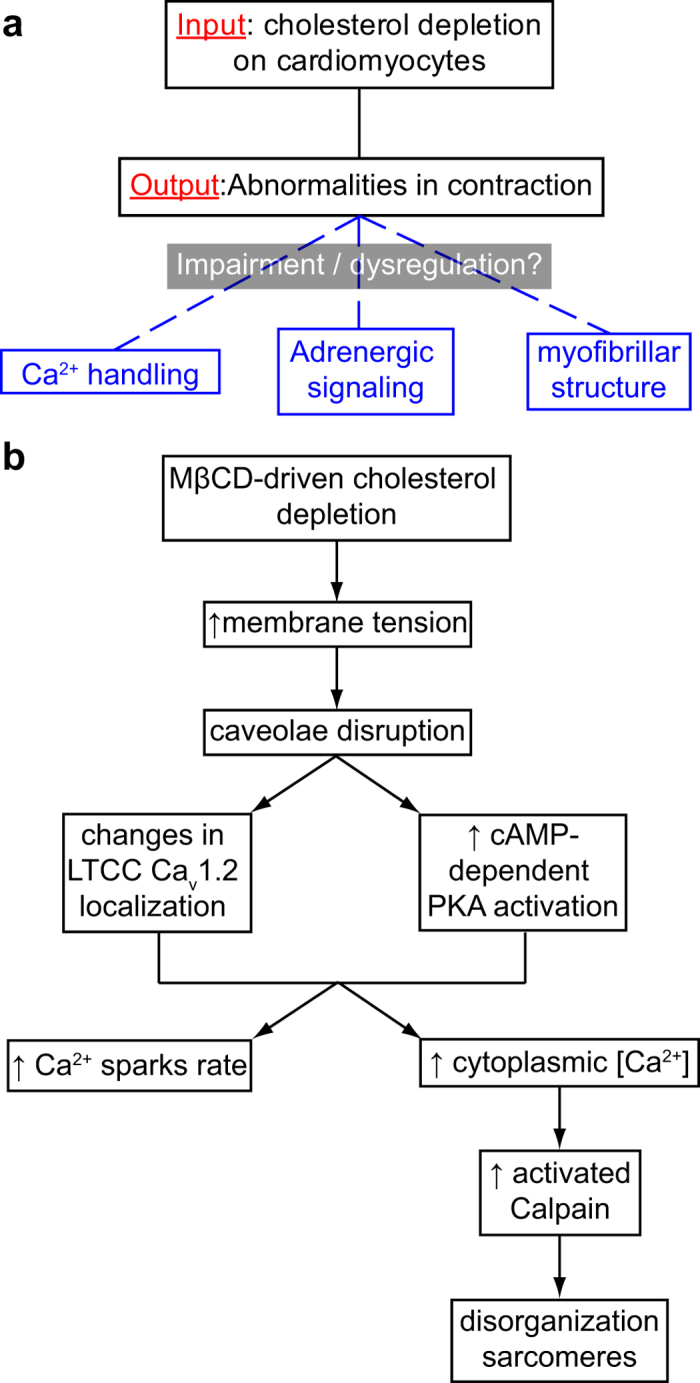
Cholesterol depletion triggers signaling cascade that culminates with contraction dynamics impairment and myofibril disruption. (**a**) Cardiomyocytes submitted to cholesterol depletion presented contraction abnormalities. The main hypotheses for that experimental output were that MβCD treatment was impairing or deregulating (either/or) Ca^2+^ handling, adrenergic signaling or the myofibrillar architecture. (**b**) As a summary, we saw that cholesterol depletion induced an increase in membrane tension leading to disruption in cav3 (caveolar marker) membrane localization. Since caveolae organizes β-adrenergic receptors and LTCC we saw that its disruption caused changes in Ca_v_1.2 (LTCC pore subunit) localization and also activated β-adrenergic receptors since we measured an elevation in cAMP-dependent PKA activation. That raised both Ca^2+^ sparks rate and intracellular Ca^2+^ concentration. The latter increased calpain activity which ultimately led to myofibrillar architecture disorganization impairing contraction of the cardiomyocytes.

TFM experiments demonstrated that even though cardiomyocytes increased both peak and trough strain energies upon cholesterol depletion, the resultant strain energy (peak minus trough) shows a decrease in cell contractility. In addition to that, the increase in trough strain energy and decrease in overall contractility suggests impairment in cell shortening and increase in isometric force generation in low cholesterol scenarios. Interestingly, isometric cardiac contraction is correlated with myofibrillar disarray in left ventricles obtained from patients with idiopathic hypertrophic subaortic stenosis^59^. Moreover, our TFM data also showed that more rapid and less rhythmic contractions were observed in low-cholesterol scenarios. It is known that cardiomyocyte contraction is a multifactorial process that is regulated in three main interdependent levels: Ca^2+^ handling, adrenergic signaling cascade, sarcomeric integrity and caveolae domains are thought to be important to all of them (Fig. 7).

Caveolae are flask-shaped and cholesterol-enriched plasma membrane invaginations that regulate many cellular processes including lipid homeostasis and adaptation to membrane tension^47^,^60^,^61^. Caveolins are proteins that are responsible not only for scaffolding caveolae but also are important for cell signaling^62^ and caveolin-3 is the most abundant isoform in striated muscle^10^,^63^. By doing immunostaining we saw that cholesterol depletion changed drastically the distribution of caveolin-3 in the cardiomyocyte plasma membranes with the control cells having a more punctate distribution of caveolin-3 along the myofibers whereas MβCD treated cells showed a disperse labeling of that protein. This result can also be corroborated by the increase in membrane tension that we saw on our OT-tether pulling experiments since cells tend to disassemble caveolae under mechanical stress^61^.

Calcium is one of the three main pillars that regulate cardiomyocyte contraction. For the neonatal cardiomyocyte case, extracellular Ca^2+^ plays a fundamental role in contraction^44^,^48^,^49^,^50^,^51^,^64^ since those cells lack T-tubules^11^. T-tubules are membranous cisternae networks that couple extracellular Ca^2+^ entry to calcium-induced-calcium-release that is essential for adult ventricular cardiomyocytes contraction^65^,^66^. Since neonatal heart cells depend mostly on extracellular Ca^2+^ to contract (revised by ref. 67) and we were doing perturbations in the plasma membrane cholesterol we sought to investigate whether those perturbations were affecting Ca^2+^ channels, membrane-bound Ca^2+^ channel regulators and Ca^2+^ influx.

Some members of the G-protein Coupled Receptors (GPCRs) family, known as adrenergic receptors (β1 and β2-ARs, e.g), regulate cardiac Ca^2+^ signaling, at the plasma membrane level. Upon agonist binding to the β-ARs, adenylyl-cyclase (AC) is activated and accelerates the production of cAMP, which in turn activates PKA. PKA phosphorylates the pore-forming subunit of LTCC, Ca_v_1.2, promoting opening of the channel and Ca^2+^ entry. It had been shown via immunoprecipitation that caveolin-3, Ca_v_1.2, β2-AR (but not β1-AR), AC and PKA are closely associated in neonatal murine cardiomyocytes^18^ and it is also established that caveolin binding inhibits AC activity^68^. Experiments performed in murine neonatal cardiomyocytes show that Filipin, a polyene antibiotic that binds to unsterified cholesterol and disorganizes caveolae domains, increased by a factor of 2 the β2-AR stimulated myocyte contraction rate upon isoproterenol incubation^69^. There is also evidence that 1% hydroxypropyl-β-cyclodextrin treatment increased cAMP accumulation in rat neonatal cardiomyocyes upon agonist binding (isoproterenol, zinterol or forskolin)^70^. These two above-mentioned studies increase the body of evidence that demonstrates that caveolar localization dampens β2-AR signaling. Our immunostaining results show that when the cells are submitted to cholesterol depletion, Ca_v_1.2 subunit of LTCC changes distribution within the cells being excluded from the perinuclear region. We also demonstrated, by enzymatic assay, that cAMP-dependent PKA activity increases when cholesterol is depleted even in the absence of β2-AR agonists, which might increase the number of Ca_v_1.2 opening events leading to increase of Ca^2+^ influx. In fact, we saw that cholesterol reduction led to an increase in the Ca^2+^ probe fluorescence in the cytoplasm of the cardiomyocytes. Moreover, the Ca^2+^ spikes were more frequent and less synchronized in the MβCD treated cells, which correlates to our TFM results and corroborates our hypothesis that cholesterol sequestration impairs both contraction dynamics and Ca^2+^ handling by the cardiac cells.

The last pillar that governs cardiac contraction is sarcomeric integrity. Since we measured impairment in cardiomyocyte contraction dynamics and also changes in membrane tension in those cells we hypothesized that MβCD treatment was somehow perturbing the sarcomeric actin cytoskeleton that plays an important role in cardiomyocyte contraction^71^. By doing actin and α-actinin staining we demonstrated that cholesterol chelated cells showed sarcomeres that were not equally spaced throughout the myofibers with curvy z-bands as opposed to the control cells that show equally spaced and straight z-bands. Image analysis showed that, on average, z-bands get closer and the average angles between z-bands get bigger when cholesterol is removed. Impairment in myofibrillar architecture can also be correlated with decrease in cell contractility and defects in cell relaxation in cholesterol depleted cardiomyocytes, as observed in our TFM experiments. It is suggestive that impairment in cardiomyocyte shortening might give rise to myofibrillar disarray, as previously observed^59^.

In addition, we decided to investigate whether the disruption of the myofibers was caused by Ca^2+^-dependent proteolytic activity in the sarcoplasm. In order to test that, we measured cytoplasmic calpain enzymatic activity. It is known that cytoplasmic Ca^2+^ triggers calpain activation which in turn can degrade sarcomeric proteins such as troponins, tropomyosins and titins leading to abrogation of the z-bands organization^28^. Since we saw an increase in cytoplasmic Ca^2+^ upon cholesterol depletion we hypothesized that it might be activating calpain degradation of myofibers impacting the normal contraction behavior of those cells. In fact, we saw an increase in calpain activity due to cholesterol depletion and that correlates with the disorganization of the z-bands that we measured.

Overall this work points out to a new mechanism in which sarcoplasmic cholesterol depletion by MβCD incubation leads to impairment in cardiomyocyte contractility and calcium handling increasing cortical mechanical properties values, cytoplasmic Ca^2+^ concentration, calpain proteolytic activation and disorganization of myofibers.

## Additional Information

**How to cite this article:** Hissa, B. *et al*. Cholesterol depletion impairs contractile machinery in neonatal rat cardiomyocytes. *Sci. Rep.* **7**, 43764; doi: 10.1038/srep43764 (2017).

**Publisher's note:** Springer Nature remains neutral with regard to jurisdictional claims in published maps and institutional affiliations.

## Supplementary Material

Supplementary Information

## Figures and Tables

**Table 1:** Membrane tension σ and bending rigidity κ for cardiomyocytes (mean ± standard error).

Condition	σ (pN.μm^−1^)	κ (pN.μm)
Control	82 ± 10	0.19 ± 0.02
MβCD 5.0 mmol/L	97 ± 13	0.27 ± 0.03
MβCD 7.5 mmol/L	105 ± 17	0.35 ± 0.06

## References

[b1] Simons K. & Ikonen E. Functional rafts in cell membranes. Nature 387, 569–572, doi: 10.1038/42408 (1997). 9177342

[b2] Kwik J. . Membrane cholesterol, lateral mobility, and the phosphatidylinositol 4,5-bisphosphate-dependent organization of cell actin. Proceedings of the National Academy of Sciences of the United States of America 100, 13964–13969, doi: 10.1073/pnas.2336102100 (2003). 14612561 PMC283529

[b3] Byfield F. J., Aranda-Espinoza H., Romanenko V. G., Rothblat G. H. & Levitan I. Cholesterol depletion increases membrane stiffness of aortic endothelial cells. Biophysical journal 87, 3336–3343, doi: 10.1529/biophysj.104.040634 (2004). 15347591 PMC1304801

[b4] Hissa B. . Membrane cholesterol removal changes mechanical properties of cells and induces secretion of a specific pool of lysosomes. PloS one 8, e82988–e82988 (2013). 24376622 10.1371/journal.pone.0082988 PMC3869752

[b5] Sun M. . The effect of cellular cholesterol on membrane-cytoskeleton adhesion. Journal of cell science 120, 2223–2231, doi: 10.1242/jcs.001370 (2007). 17550968

[b6] Tanaka S. . Mechanism of Statin-Induced Contractile Dysfunction in Rat Cultured Skeletal Myofibers. Journal of Pharmacological Sciences 114, 454–463, doi: 10.1254/jphs.10229FP (2010). 21127387

[b7] Sakamoto K., Honda T., Yokoya S., Waguri S. & Kimura J. Rab-small GTPases are involved in fluvastatin and pravastatin-induced vacuolation in rat skeletal myofibers. Faseb Journal 21, 4087–4094, doi: 10.1096/fj.07-8713com (2007). 17634390

[b8] Vega-Moreno J. . Cholesterol Depletion Uncouples beta-dystroglycans from Discrete Sarcolemmal Domains, Reducing the Mechanical Activity of Skeletal Muscle. Cellular Physiology and Biochemistry 29, 905–918, doi: 10.1159/000186933 (2012). 22613990

[b9] Harvey R. D. & Calaghan S. C. Caveolae create local signalling domains through their distinct protein content, lipid profile and morphology. J Mol Cell Cardiol 52, 366–375, doi: 10.1016/j.yjmcc.2011.07.007 (2012). 21782827 PMC4120829

[b10] Song K. S. . Expression of caveolin-3 in skeletal, cardiac, and smooth muscle cells - Caveolin-3 is a component of the sarcolemma and co-fractionates with dystrophin and dystrophin-associated glycoproteins. Journal of Biological Chemistry 271, 15160–15165 (1996). 8663016 10.1074/jbc.271.25.15160

[b11] Haddock P. S. . Subcellular Ca2+ (i) gradients during excitation-contraction coupling in newborn rabbit ventricular myocytes. Circulation Research 85, 415–427 (1999). 10473671 10.1161/01.res.85.5.415

[b12] Huang H., Bae C., Sachs F. & Suchyna T. M. Caveolae Regulation of Mechanosensitive Channel Function in Myotubes. Plos One 8, doi: 10.1371/journal.pone.0072894 (2013). PMC3758351 24023653

[b13] Lo H. P. . The caveolin-cavin system plays a conserved and critical role in mechanoprotection of skeletal muscle. Journal of Cell Biology 210, 833–849, doi: 10.1083/jcb.201501046 (2015). 26323694 PMC4555827

[b14] Kozera L., White E. & Calaghan S. Caveolae act as membrane reserves which limit mechanosensitive I(Cl,swell) channel activation during swelling in the rat ventricular myocyte. PLoS One 4, e8312, doi: 10.1371/journal.pone.0008312 (2009). 20011535 PMC2788708

[b15] Maguy A., Hebert T. E. & Nattel S. Involvement of lipid rafts and caveolae in cardiac ion channel function. Cardiovascular Research 69, 798–807, doi: 10.1016/j.cardiores.2005.11.013 (2006). 16405931

[b16] Wright P. T. . Caveolin-3 regulates compartmentation of cardiomyocyte beta2-adrenergic receptor-mediated cAMP signaling. Journal of Molecular and Cellular Cardiology 67, 38–48, doi: 10.1016/j.yjmcc.2013.12.003 (2014). 24345421 PMC4266930

[b17] Calaghan S., Kozera L. & White E. Compartmentalisation of cAMP-dependent signalling by caveolae in the adult cardiac myocyte. Journal of Molecular and Cellular Cardiology 45, 88–92, doi: 10.1016/j.yjmcc.2008.04.004 (2008). 18514221

[b18] Balijepalli R. C., Foell J. D., Hall D. D., Hell J. W. & Kamp T. J. Localization of cardiac L-type Ca(2+) channels to a caveolar macromolecular signaling complex is required for beta(2)-adrenergic regulation. Proc Natl Acad Sci USA 103, 7500–7505 (2006). 16648270 10.1073/pnas.0503465103 PMC1564282

[b19] Kamp T. J. & Hell J. W. Regulation of cardiac L-type calcium channels by protein kinase A and protein kinase C. Circulation Research 87, 1095–1102 (2000). 11110765 10.1161/01.res.87.12.1095

[b20] Hulme J. T., Lin T. W., Westenbroek R. E., Scheuer T. & Catterall W. A. Beta-adrenergic regulation requires direct anchoring of PKA to cardiac CaV1.2 channels via a leucine zipper interaction with A kinase-anchoring protein 15. Proc Natl Acad Sci USA 100, 13093–13098, doi: 10.1073/pnas.2135335100 (2003). 14569017 PMC240750

[b21] Flucher B. E. & FranziniArmstrong C. Formation of junctions involved in excitation-contraction coupling in skeletal and cardiac muscle. Proceedings of the National Academy of Sciences of the United States of America 93, 8101–8106, doi: 10.1073/pnas.93.15.8101 (1996). 8755610 PMC38882

[b22] Rybin V. O., Pak E., Alcott S. & Steinberg S. F. Developmental changes in beta2-adrenergic receptor signaling in ventricular myocytes: the role of Gi proteins and caveolae microdomains. Mol Pharmacol 63, 1338–1348, doi: 10.1124/mol.63.6.1338 (2003). 12761344

[b23] Skwarek-Maruszewska A., Hotulainen P., Mattila P. K. & Lappalainen P. Contractility-dependent actin dynamics in cardiomyocyte sarcomeres. Journal of cell science 122, 2119–2126, doi: 10.1242/jcs.046805 (2009). 19470580

[b24] Squire J. M. Architecture and function in the muscle sarcomere. Current Opinion in Structural Biology 7, 247–257, doi: 10.1016/s0959-440x(97)80033-4 (1997). 9094325

[b25] Sanger J. W. . How to build a myofibril. Journal of Muscle Research and Cell Motility 26, 343–354, doi: 10.1007/s10974-005-9016-7 (2005). 16465476

[b26] Asp M. L., Martindale J. J., Heinis F. I., Wang W. & Metzger J. M. Calcium mishandling in diastolic dysfunction: Mechanisms and potential therapies. Biochimica Et Biophysica Acta-Molecular Cell Research 1833, 895–900, doi: 10.1016/j.bbamcr.2012.09.007 (2013). PMC3586938 23022395

[b27] Matsumura Y. . Intracellular calcium level required for calpain activation in a single myocardial cell. J Mol Cell Cardiol 33, 1133–1142, doi: 10.1006/jmcc.2001.1373 (2001). 11444918

[b28] Portbury A. L., Willis M. S. & Patterson C. Tearin’ Up My Heart: Proteolysis in the Cardiac Sarcomere. Journal of Biological Chemistry 286, 9929–9934, doi: 10.1074/jbc.R110.170571 (2011). 21257759 PMC3060546

[b29] Christian A. E., Haynes M. P., Phillips M. C. & Rothblat G. H. Use of cyclodextrins for manipulating cellular cholesterol content. Journal of lipid research 38, 2264–2272 (1997). 9392424

[b30] Kavalipati N., Shah J., Ramakrishan A. & Vasnawala H. Pleiotropic effects of statins. Indian J Endocrinol Metab 19, 554–562, doi: 10.4103/2230-8210.163106 (2015). 26425463 PMC4566334

[b31] Yancey P. G. . Cellular cholesterol effect mediated by cyclodextrins - Demonstration of kinetic pools and mechanism of efflux. Journal of Biological Chemistry 271, 16026–16034 (1996). 8663188 10.1074/jbc.271.27.16026

[b32] Irie T., Fukunaga K., Garwood M. K., Carpenter T. O. & Pitha J. Hydroxypropylcyclodextrins in parenteral use.2. Effects on transport and disposition of lipids in rabbit and humans. Journal of Pharmaceutical Sciences 81, 524–528, doi: 10.1002/jps.2600810610 (1992). 1522488

[b33] Hissa B. . Membrane cholesterol regulates lysosome-plasma membrane fusion events and modulates Trypanosoma cruzi invasion of host cells. PLoS neglected tropical diseases 6, e1583–e1583, doi: 10.1371/journal.pntd.0001583 (2012). 22479662 PMC3313932

[b34] Oakes P. W., Beckham Y., Stricker J. & Gardel M. L. Tension is required but not sufficient for focal adhesion maturation without a stress fiber template. Journal of Cell Biology 196, 363–374, doi: 10.1083/jcb.201107042 (2012). 22291038 PMC3275371

[b35] Aratyn-Schaus Y., Oakes P. W., Stricker J., Winter S. P. & Gardel M. L. Preparation of complaint matrices for quantifying cellular contraction. Journal of visualized experiments: JoVE, doi: 10.3791/2173 (2010). PMC3159639 21178972

[b36] Maruthamuthu V. & Gardel M. L. Protrusive Activity Guides Changes in Cell-Cell Tension during Epithelial Cell Scattering. Biophysical Journal 107, 555–563, doi: 10.1016/j.bpj.2014.06.028 (2014). 25099795 PMC4129477

[b37] Sabass B., Gardel M. L., Waterman C. M. & Schwarz U. S. High resolution traction force microscopy based on experimental and computational advances. Biophysical Journal 94, 207–220, doi: 10.1529/biophysj.107.113670 (2008). 17827246 PMC2134850

[b38] Plotnikov S. V., Sabass B., Schwarz U. S. & Waterman C. M. High-Resolution Traction Force Microscopy. Quantitative Imaging in Cell Biology 123, 367–394, doi: 10.1016/b978-0-12-420138-5.00020-3 (2014). PMC4699589 24974038

[b39] Oakes P. W., Banerjee S., Marchetti M. C. & Gardel M. L. Geometry Regulates Traction Stresses in Adherent Cells. Biophysical Journal 107, 825–833, doi: 10.1016/j.bpj.2014.06.045 (2014). 25140417 PMC4142236

[b40] Butler J. P., Tolic-Norrelykke I. M., Fabry B. & Fredberg J. J. Traction fields, moments, and strain energy that cells exert on their surroundings. American Journal of Physiology-Cell Physiology 282, C595–C605 (2002). 11832345 10.1152/ajpcell.00270.2001

[b41] Pontes B. . Cell cytoskeleton and tether extraction. Biophysical journal 101, 43–52, doi: 10.1016/j.bpj.2011.05.044 (2011). 21723813 PMC3127177

[b42] Pontes B. . Membrane Elastic Properties and Cell Function. Plos One 8, doi: 10.1371/journal.pone.0067708 (2013). PMC3701085 23844071

[b43] Guatimosim S., Guatimosim C. & Song L.-S. Imaging Calcium Sparks in Cardiac Myocytes. Light Microscopy: Methods and Protocols 689, 205–214, doi: 10.1007/978-1-60761-950-5_12 (2011). PMC3233356 21153794

[b44] Gomez J. P., Potreau D. & Raymond G. Intracellular calcium transients from newborn rat cardiomyocytes in primary culture. Cell Calcium 15, 265–275, doi: 10.1016/0143-4160(94)90066-3 (1994). 8055543

[b45] Koster G., Cacciuto A., Derenyi I., Frenkel D. & Dogterom M. Force barriers for membrane tube formation. Physical Review Letters 94, doi: 10.1103/PhysRevLett.94.068101 (2005). 15783778

[b46] Echarri A. . Caveolar domain organization and trafficking is regulated by Abl kinases and mDia1. J Cell Sci 125, 3097–3113, doi: 10.1242/jcs.090134 (2012). 22454521

[b47] Echarri A. & Del Pozo M. A. Caveolae - mechanosensitive membrane invaginations linked to actin filaments. Journal of Cell Science 128, 2747–2758, doi: 10.1242/jcs.153940 (2015). 26159735

[b48] Seki S. . Fetal and postnatal development of Ca2+ transients and Ca2+ sparks in rat cardiomyocytes. Cardiovascular Research 58, 535–548, doi: 10.1016/s0008-6363(03)00255-4 (2003). 12798426

[b49] Escobar A. L. . Developmental changes of intracellular Ca2+ transients in beating rat hearts (vol 286, pg H972, 2004). American Journal of Physiology-Heart and Circulatory Physiology 287, H433–H433, doi: 10.1152/ajpheart.00246.2004 (2004). 14644760

[b50] Korhonen T., Hanninen S. L. & Tavi P. Model of Excitation-Contraction Coupling of Rat Neonatal Ventricular Myocytes. Biophysical Journal 96, 1189–1209, doi: 10.1016/j.bpj.2008.10.026 (2009). 19186154 PMC2716686

[b51] Hamaguchi S. . Developmental Changes in Excitation-Contraction Mechanisms of the Mouse Ventricular Myocardium as Revealed by Functional and Confocal Imaging Analyses. Journal of Pharmacological Sciences 123, 167–175, doi: 10.1254/jphs.13099FP (2013). 24096881

[b52] Weiss S., Oz S., Benmocha A. & Dascal N. Regulation of cardiac L-type Ca(2)(+) channel CaV1.2 via the beta-adrenergic-cAMP-protein kinase A pathway: old dogmas, advances, and new uncertainties. Circ Res 113, 617–631, doi: 10.1161/circresaha.113.301781 (2013). 23948586

[b53] Bodi I., Mikala G., Koch S. E., Akhter S. A. & Schwartz A. The L-type calcium channel in the heart: the beat goes on. J Clin Invest 115, 3306–3317, doi: 10.1172/jci27167 (2005). 16322774 PMC1297268

[b54] Harvey R. D. & Hell J. W. CaV1.2 signaling complexes in the heart. J Mol Cell Cardiol 58, 143–152, doi: 10.1016/j.yjmcc.2012.12.006 (2013). 23266596 PMC3628296

[b55] Maxfield F. R. & Tabas I. Role of cholesterol and lipid organization in disease. Nature 438, 612–621, doi: 10.1038/nature04399 (2005). 16319881

[b56] Babiychuk E. B. . Membrane cholesterol regulates smooth muscle Phasic contraction. Journal of Membrane Biology 198, 95–101, doi: 10.1007/s00232-004-0663-1 (2004). 15138749

[b57] Baines A. J., Pinder J. C. & Fordham J. The spectrin-associated cytoskeleton in mammalian heart. Frontiers in Bioscience 10, 3020–3033, doi: 10.2741/1759 (2005). 15970557

[b58] Kilsdonk E. P. . Cellular cholesterol efflux mediated by cyclodextrins. The Journal of biological chemistry 270, 17250–17256, doi: 10.1074/jbc.270.29.17250 (1995). 7615524

[b59] Bulkley B. H., Weisfeldt M. L. & Hutchins G. M. Isometric cardiac contraction. a possible cause of the disorganized myocardial pattern of idiopathic hypertrophic subaortic stenosis. N Engl J Med 296, 135–139, doi: 10.1056/nejm197701202960303 (1977). 556638

[b60] Parton R. G. & del Pozo M. A. Caveolae as plasma membrane sensors, protectors and organizers. Nature Reviews Molecular Cell Biology 14, 98–112, doi: 10.1038/nrm3512 (2013). 23340574

[b61] Sinha B. . Cells Respond to Mechanical Stress by Rapid Disassembly of Caveolae. Cell 144, 402–413, doi: 10.1016/j.cell.2010.12.031 (2011). 21295700 PMC3042189

[b62] Shaul P. W. & Anderson R. G. W. Role of plasmalemmal caveolae in signal transduction. American Journal of Physiology-Lung Cellular and Molecular Physiology 275, L843–L851 (1998). 10.1152/ajplung.1998.275.5.L843 9815100

[b63] Tang Z. L. . Molecular cloning of caveolin-3, a novel member of the caveolin gene family expressed predominantly in muscle. Journal of Biological Chemistry 271, 2255–2261 (1996). 8567687 10.1074/jbc.271.4.2255

[b64] Cohen N. M. & Lederer W. J. Changes in the calcium current of rat heart ventricular myocytes during development. J Physiol 406, 115–146 (1988). 2855434 10.1113/jphysiol.1988.sp017372 PMC1191091

[b65] Bers D. M. Cardiac excitation-contraction coupling. Nature 415, 198–205, doi: 10.1038/415198a (2002). 11805843

[b66] Bers D. M. & Guo T. Calcium signaling in cardiac ventricular myocytes. Ann N Y Acad Sci 1047, 86–98, doi: 10.1196/annals.1341.008 (2005). 16093487

[b67] Louch W. E., Koivumaki J. T. & Tavi P. Calcium signalling in developing cardiomyocytes: implications for model systems and disease. Journal of Physiology-London 593, 1047–1063, doi: 10.1113/jphysiol.2014.274712 (2015). PMC4358669 25641733

[b68] Toya Y. . Inhibition of adenylyl cyclase by caveolin peptides. Journal of Hypertension 16, S79–S79 (1998). 10.1210/endo.139.4.5957 9528990

[b69] Xiang Y., Rybin V. O., Steinberg S. F. & Kobilka B. Caveolar localization dictates physiologic signaling of beta(2)-adrenoceptors in neonatal cardiac myocytes. Journal of Biological Chemistry 277, 34280–34286, doi: 10.1074/jbc.M201644200 (2002). 12097322

[b70] Rybin V. O., Xu X. H., Lisanti M. P. & Steinberg S. F. Differential targeting of beta-adrenergic receptor subtypes and adenylyl cyclase to cardiomyocyte caveolae - A mechanism to functionally regulate the cAMP signaling pathway. Journal of Biological Chemistry 275, 41447–41457, doi: 10.1074/jbc.M006951200 (2000). 11006286

[b71] Sequeira V., Nijenkamp L. L., Regan J. A. & van der Velden J. The physiological role of cardiac cytoskeleton and its alterations in heart failure. Biochim Biophys Acta 1838, 700–722, doi: 10.1016/j.bbamem.2013.07.011 (2014). 23860255

