# Supplementary material for: Cholesterol depletion impairs contractile machinery in neonatal rat cardiomyocytes
Source: Sci Rep. 2017 Mar 3;7:43764. doi: 10.1038/srep43764 (PMC5335656; doi:10.1038/srep43764)
Supplement: Supplementary Information [file srep43764-s1.pdf]

SUPPLEMENTAL MATERIAL FROM:

Cholesterol depletion impairs contractile machinery in neonatal rat cardiomyocytes

Barbara Hissa[1*]; Patrick W. Oakes[1], Bruno Pontes[2], Guillermina Ramírez-San Juan[1]; and Margaret L. Gardel[1*]

[1] James Franck Institute, Institute for Biophysical Dynamics and Physics Department, University of Chicago, Chicago, IL, United States

[2] LPO-COPEA, Instituto de Ciências Biomédicas, Universidade Federal do Rio de Janeiro, Rio de Janeiro, RJ, Brazil

*** Corresponding authors**

Supplemental figures and figure legends

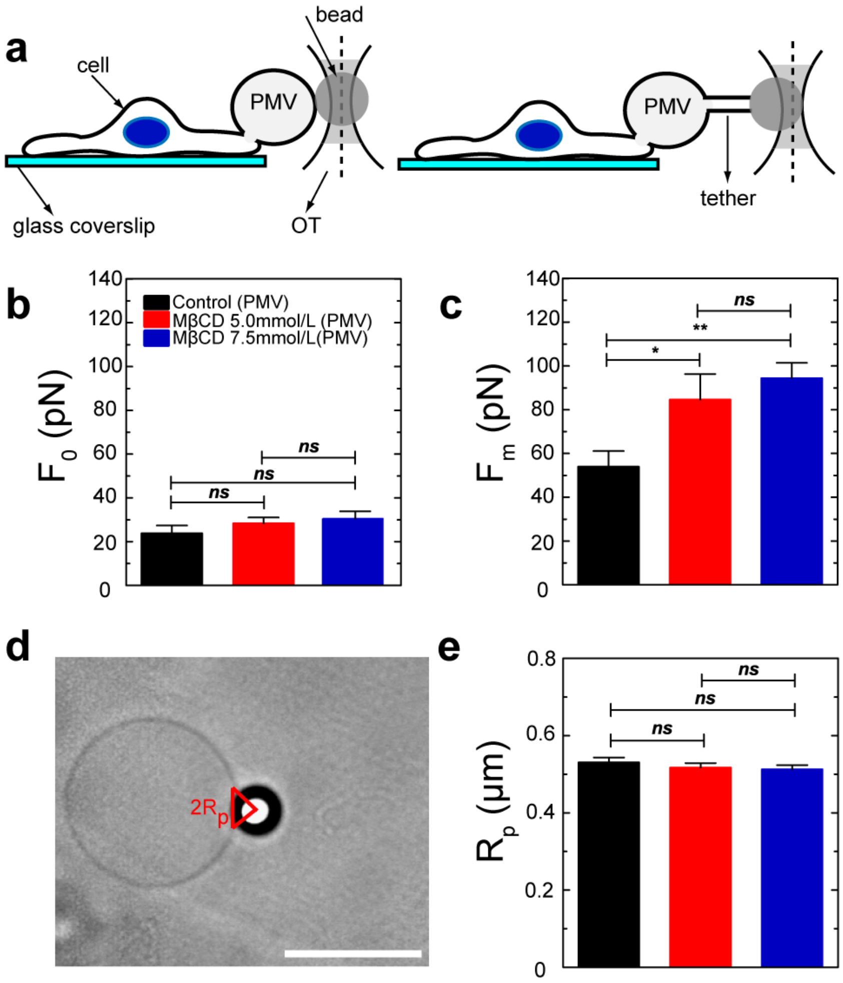

Figure S1: **Cholesterol depletion changes membrane tension but not bending rigidity of cardiomyocyte plasma membrane vesicles** (A) Cartoon of a cardiomyocyte and an optically-trapped polystyrene bead attached to the pre-formed plasma membrane vesicle PMV (left) and formation of the tether tube (right). (B) Mean and standard error for F_0 values of control (n=15) and cholesterol depleted cardiomyocytes treated with either 5.0 (n=18) or 7.5 mmol/L MβCD (n=16). (C) Mean and standard error for F_m values of control (n=15) and cholesterol depleted cardiomyocytes treated with either 5.0 (n=18) or 7.5 mmol/L MβCD (n=16). (D) Bright field image of a real cardiomyocyte PMV showing the schematics for calculating the patch radius (R_p). Scale bar 10 μm. (E) Mean and standard error for R_p values of control (n=15) and cholesterol depleted cardiomyocytes treated with either 5.0 (n=17) or 7.5 mmol/L MβCD (n=16). ** p<0.05, ns= not statistically different according Students T test.

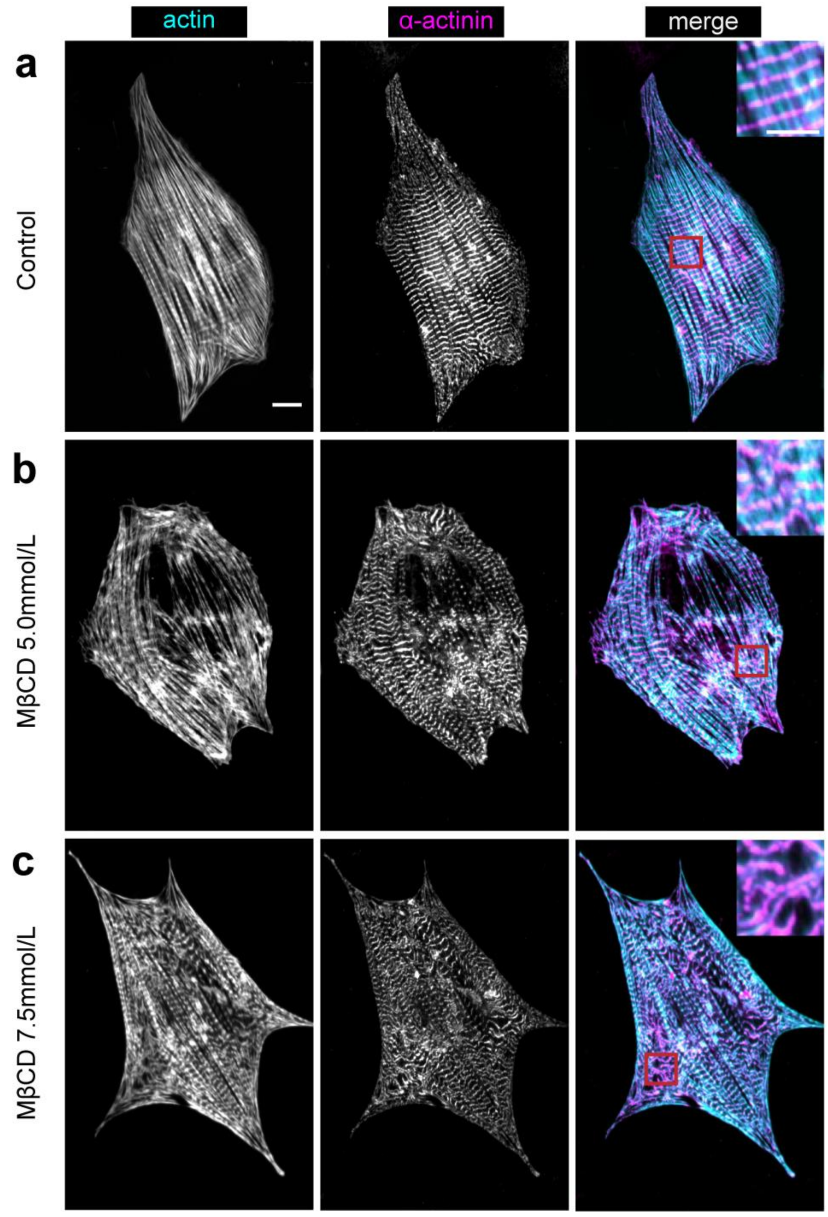

<u>Figure S2</u>: Cholesterol depletion changes myofibril architecture of primary neonatal cardiomyocytes. Representative images of control (A), MβCD 5.0 mmol/L (B) and MβCD 7.5 mmol/L treated cells (C) fixed and labeled for actin (Phalloidin) (cyan) (right panel) and α-actinin (magenta) (mid panel). Merges of the two channels are shown in the left panel. Insets show merged images of actin and α-actinin. Scale bar 10 μm.

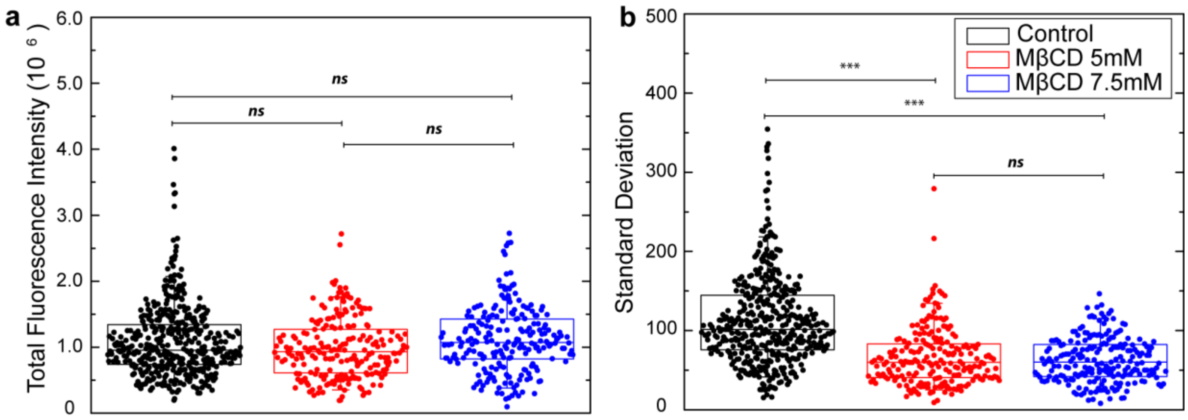

<u>Figure S3:</u> Cholesterol depletion changes caveolin-3 distribution in neonatal cardiomyocytes. (A) Box plots showing background corrected total fluorescence intensity measured for 5 distinct boxes/ cell distributed uniformly across cell area. At least 20 cells/ condition were analyzed. The total fluorescence is a measure of the total amount of caveolin-3 within that particular region. No statistical differences were found between analyzed groups. (B) Standard deviation of the mean obtained for the same regions analyzed in (A). The standard deviation informs how uniformly distributed caveolin-3 is within each analyzed region. Statistical differences were found between control and cholesterol depleted groups. Cholesterol depleted groups have a more uniformly distributed fluorescence pattern in comparison to control cells and that corroborates what is depicted in Fig.4. *** $p<0.0001$, ns= not statistically different according to Student's T-test.

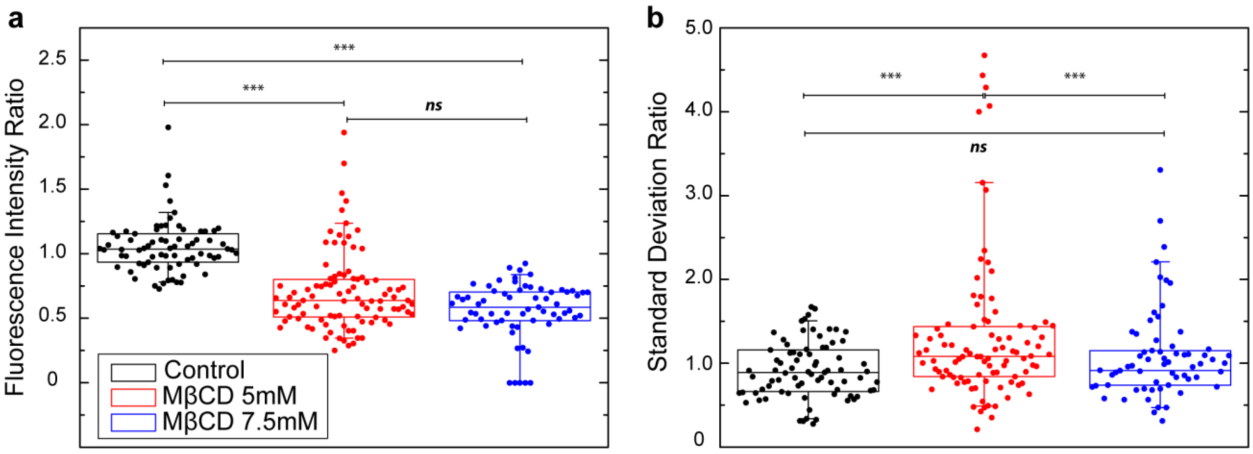

<u>Figure S4</u>: Cholesterol depletion changes distribution of $Ca_v1.2$ subunit of L-type Ca^{2+} channel. (A) Boxplots showing the ratio between background corrected total fluorescence for 5 distinct boxes/ cell distributed around the perinuclear region and for 5 different boxes that were placed away from the perinuclear region. At least 20 cells/ condition were analyzed. There is a significant reduction in the fluorescence ratio when cholesterol is depleted. This result shows that cholesterol depletion redistributes $Ca_v1.2$ across the cell membrane away from the perinuclear region. (B) Standard deviation of the mean obtained for the same regions analyzed in (A). The values for standard deviation increased for 5mM MβCD and showed a tendency for increasing for 7.5mM MβCD case. This result reveals that cholesterol depletion is changing the distribution of $Ca_v1.2$ across the cell and that distribution is becoming less uniform. *** $p<0.0001$, ns= not statistically different according to Student's T-test.

Supplemental tables:

Table S1: Summary of values obtained for cardiomyocytes PMVs

Condition	F_0 (pN)	F_m (pN)	R_p (µm)	R (µm)	σ (pN.µm^{-1})	κ (pN.µm)
**Control (PMV)**	24 ± 4	54 ± 7	0.53 ± 0.01	0.21 ± 0.07	9.0 ± 3	0.8 ± 0.3
**MβCD 5.0 mmol/L (PMV)**	28 ± 3	85 ± 12	0.52 ± 0.01	0.13 ± 0.03	18 ± 5	0.6 ± 0.2
**MβCD 7.5 mmol/L (PMV)**	30 ± 3	94 ± 7	0.51 ± 0.01	0.12 ± 0.02	20 ± 4	0.6 ± 0.1

Detailed methods

Primary culture of neonatal rat cardiomyocytes

Ten to fifteen rat pups, Wistar strain, were euthanized and had their hearts removed aseptically, immediately transferred to an ice-cold Hank's Balanced Salt Solution (Sigma-Aldrich, St. Louis, MO) (pH 7.4) and kept on ice until the mechanical dissociation step. Next, the hearts were rinsed in fresh cold HBSS, minced into 1 mm^3 fragments and partially digested in an enzymatic solution containing HBSS and 1% (vol/vol) Trypsin-EDTA 0.25% (Corning, New York, NY) overnight, at 4^oC, under gentle agitation. The following day, 1 mL of soybean trypsin inhibitor (Sigma-Aldrich; 1 mg/mL in HBSS) was added to the solution. Type-II Collagenase (Worthington, Lakewood NJ; 1mg/mL), was also added to the semi-digested hearts and incubated with the cardiac tissue for approximately 40 minutes, at 37^oC under constant rocking. Tissue clumps were mechanically dissociated with a plastic sterile pipette and the resultant cell solution was filtered through a 70 µm cell strainer (BD Bioscience, San Jose, CA) and centrifuged at 300 g for 5 minutes. The cell pellet was resuspended in high-glucose DMEM (Corning), supplemented with 10% (vol/vol) fetal bovine serum (FBS) and 1% (vol/vol) penicillin/streptomycin (100 U/mL/100 µg/mL; Corning). The cell suspension was pre-plated for 2 hours at 37°C in a 5% CO_2 incubator in order to remove non-muscle cells. Purified cardiomyocytes were collected, seeded at a density of 4.0×10^5 cells/well onto 6-well plates containing round coverslips coated with fibronectin alone or containing fibronectin crosslinked to polyacrylamide (PAA) gels for traction force microscopy experiments. Cells were kept at 37°C in a humidified incubator, 5% CO_2, for 72 hours before experimental procedures. New cultures were prepared for each experiment.

Drug treatments

In order to deplete cholesterol from the cardiomyocytes plasma membranes, the cells were rinsed 3x with PBS containing calcium and magnesium (PBS +/+; Corning) and incubated with either 5.0 or 7.5 mM methyl-beta cyclodextrin (MβCD; Sigma-Aldrich), dissolved in serum free DMEM, for 45 minutes, at 37°C. After the incubation period, cells were rinsed again 3x with PBS +/+ and fresh serum free media was added.

Immunofluorescence

Following treatment, the cardiomyocytes were rinsed in warm cytoskeleton buffer (CB buffer; 10 mM MES, 3 mM $MgCl_2$, 1.38 M KCl, and 20 mM EGTA) and then fixed and permeabilized in 4% PFA (Electron Microscopy Sciences, Hatfield, PA), 1.5% bovine serum albumin (BSA; Thermo Fisher Scientific, Waltham, MA), and 0.5% Triton X-100 (Sigma- Aldrich) in CB buffer for 10 min at 37°C. Coverslips were then rinsed three times in PBS and blocked in CB buffer plus 1.5% BSA and 0.5% Triton X-100 for 30 minutes at room temperature. The primary antibodies used in this work were the following: mouse monoclonal anti-sarcomeric α-actinin (1:200; Sigma-Aldrich), rabbit polyclonal anti-caveolin 3 (1:200; Thermo-Scientific), mouse monoclonal alpha 2 subunit L-type Ca^{2+} channel (1:250; Abcam, Cambridge, MA) The primary antibodies were diluted in the same buffer used for the blocking step and the cells were incubated at room temperature for 1h. Next, the coverslips were rinsed 3x with PBS and incubated with secondary antibodies AlexaFluor 647 donkey anti-mouse (1:300; Invitrogen, Thermo Fisher Scientific, Waltham, MA) and AlexaFluor 568 goat anti-rabbit (1:300; Invitrogen). The actin cytoskeleton was labeled using Phalloidin conjugated with AlexaFluor 488 (1:400; Invitrogen). Coverslips were rinsed again in PBS and mounted on glass slides using the SlowFade Antifade kit (Invitrogen). Images of cells were taken on Ti-E Nikon inverted microscope (Nikon, Melville, NY) with a confocal scan head (CSU-X; Yokogawa Electric, Musashino, Tokyo, Japan), laser merge module containing 491, 561, and 642 laser lines (Spectral Applied Research, Richmond Hill, Ontario, Canada), and an HQ2 CCD camera (Roper Scientific, Trenton, NJ). Metamorph acquisition software (Molecular Devices, Eugene, OR) was used to control the microscope hardware. Images were acquired using either 60x 1.49 NA ApoTIRF or 40x 1.30 Plan Fluor oil-immersion objectives.

Traction Force Microscopy

For Traction Force Microscopy (TFM) experiments, cardiomyocytes were isolated and plated on polyacrylamide (PAA) substrates as previously described in Oakes *et al.* [1] and Aratyn-Schaus *et*

al.[2]. Briefly, gels were polymerized from mixtures of acrylamide/bis-acrylamide solutions to yield 8.64 KPa shear modulus gels. Gels were polymerized on top of silanized glass coverslips and contained far-red 40 nm fluorescent beads (Invitrogen) embedded to serve as deformation markers. Fibronectin (Millipore, Billerica, MA) was covalently crosslinked to the gel surface using hydrazine hydrate (Sigma-Aldrich) [3]. Methods for traction force reconstruction have been previously described in the literature [1,2,4-6]. Briefly, the fluorescent beads in the gel were imaged at a frame rate of 2.5 images/second. Following the experiment, cells were detached from the substrate using 0.5% sodium dodecyl sulfate and a reference image of the embedded fluorescent beads was also taken. Images were aligned to correct for drift, and compared with the reference image using particle imaging velocimetry software (http://www. oceanwave.jp/softwares/mpiv/) in MATLAB to produce a displacement field with a grid spacing of 1.43 mm [6]. Displacement vectors were filtered and interpolated using the Kriging interpolation method. Traction stresses were reconstructed from the displacement field via Fourier Transform Traction Cytometry [4,7], using zeroth-order regularization. The same regularization parameters were used for all datasets. Strain energy per cell area was also calculated.

Tether extraction from cardiomyocytes

Cardiomyocytes from control and cholesterol depleted samples were submitted to tether extraction using an infrared optical tweezers (OT) setup. For this assay, polystyrene beads (radius 1.52±0.02 μm, Polysciences, Warrington) were added to the culture dish containing the cardiomyocytes and the dish was placed on the microscope. The OT captured a single polystyrene bead and was used to press that bead against the surface of a chosen cardiomyocyte for 5 seconds to allow bead attachment. After bead attachment, , the automated microscope stage (Prior Scientific, Rockland, MA) was moved with a controlled and constant speed (1 μm/s). Movies were taken during the tether extraction experiment using a CCD Hamamatsu C2400 camera (Hamamatsu, Japan) coupled with a SCION FG7 frame grabber (Scion Corporation, Torrance, CA) at a 10 frames/second capture rate. The OT setup and force calibration were performed as previously described [8,9]. In order to calculate the bending modulus κ and membrane tension σ we also measured the tethers radii by using Scanning Electron Microscopy (SEM) according to previously published work [8-10].

Tether extraction from plasma membrane vesicles

In order to separate the contribution from both plasma membrane and cytoskeleton to cortical mechanical properties we decided to make plasma membrane vesicles (PMVs) as previously reported [9,11]. Briefly, after incubating the cardiomyocytes with MβCD, we rinsed the cells and exposed them to PMV solution (25mM formaldehyde, 20mM DTT, 2mM $CaCl_2$, 10mM HEPES, 0.15M NaCl, pH 7.4). Cells were kept in this solution for 30 minutes in order to make PMVs. After incubation, cells were rinsed carefully and fresh serum free DMEM was added before the samples were submitted to tether extraction using the same setup as described above.

Imaging of calcium sparks

For imaging Ca^{2+} in the cardiomyocytes, cells were incubated with Fluo-4 AM (Invitrogen) diluted in DMSO at 5 μM final concentration in Tyrode Buffer (NaCl 132 mM, KCl 4 mM, 1.8 mM $CaCl_2.2H_2O$, 1.2 mM $MgCl_2.6H_2O$, 10 mM Hepes, 5.5mM glucose, pH 7.4) for 30 minutes. After the incubation period, cells were rinsed 3x with Tyrode and kept in this buffer during experiment. To capture Fluo-4 AM signal we performed live cell imaging using 20x 0.75 NA Plan Fluo Multi-immersion objective on a Nikon Ti-E microscope with a Lumen 200 Pro Light source (Prior Scientific, Cambridge UK) and an H2Q cooled CCD camera controlled via Metamorph acquisition software at 5 images/second frame rate. In order to evaluate calcium sparks, cytoplasmic fluorescence signal values were obtained using ImageJ (National Institutes of Health, Bethesda MD). In order to convert fluorescence values into Ca^{2+} concentration we used the following equation [12]:

$$[Ca^{2+}] = \frac{KR}{\frac{K}{[Ca^{2+}]_{rest}+1} - R}$$

Where K is the dissociation constant of the Ca^{2+} dye used (for Fluo-4 AM is 345 nM, *see* Invitrogen Inc.), R is the fluorescence ratio $\frac{F'}{F'_0}$ (F_0' is the minimum fluorescence recorder for a

specific cell analysed), $[Ca^{2+}]_{rest}$ for a neonatal ventricular rat cardiomyocyte is approximately 140 nM [13].

Measurement of Protein Kinase A (PKA) enzymatic activity

To measure cAMP- mediated PKA activity, cell extracts obtained from control and cholesterol depleted cardiomyocytes were submitted to enzymatic assay using PKA activity kit (Enzo Life Sciences, Farmingdale, NY). Handling of cell extracts and preparation of the assay were performed according to the manufacturer's instructions.

Quantification of α-actinin bands alignment

Spacing of α-actinin bands was measured using a custom written MATLAB script. First a linescan averaged across 5 pixels was drawn along an actin stress fiber, and the center of each α-actinin band was determined by the position of the local maximum. The spacing between bands was measured as the distance between local maxima. The orientation of each band was determined by calculating the axis of the least second moment for a small window (11x11 pixels) centered at each band. Average z-band images were created by taking the average of these windows. The average difference in angle was determined by comparing the angle difference between neighboring z-bands. Measurements of both z-line spacing and angle were performed in at least three different myofibrils within each cell in order to take into account intrinsic variations in the myofibrils that naturally occur. At least 90 myofibrils were analyzed per condition, reflecting 30 different cells from 3 independent experiments. In order to minimize the variations in shape while characterizing myofibril architecture, we chose to analyze myofibrils that were straight for at least 10 µm and away from the cell boundary.

Quantification of calpain activity

Control and cholesterol depleted cardiomyocytes were harvested and cell extracts were submitted to calpain enzymatic activity using Calpain Activity Fluorometric Assay Kit (BioVision Inc., Milpitas, CA). Cell extract handling and enzymatic activity were performed according to manufacturer's instructions.

Fourier analysis of contraction and calcium waves

Frequency analysis of calcium and contraction signals was performed in MATLAB. The signal was first baseline subtracted and the power spectrum was obtained by taking the 1D Fast Fourier Transform and squaring the result. The peak frequency was identified as the local maximum with the strongest signal in the power spectrum.

Fluorescence quantification

For measuring differences in caveolin-3 distribution between control and cholesterol depleted groups, we measured background corrected total fluorescence intensity for 5 distinct boxes per cell distributed uniformly across the cell area. At least 20 cells/ condition were analyzed. The total fluorescence is a measure of the total amount of caveolin-3 within that particular region. In order to measure how homogeneous the caveolin-3 distribution gets after cholesterol depletion we calculated the standard deviation of the mean for the same regions described previously. The lower the standard deviation values the less heterogeneous the protein distribution becomes. For measuring differences in distribution of the $Ca_v1.2$ subunit of LTCC between perinuclear regions and the rest of the cell, upon cholesterol depletion, we calculated the ratio between background corrected total fluorescence intensity measured for 5 distinct boxes near the perinuclear region and for 5 other boxes away from the perinuclear region. The ratio values decrease if $Ca_v1.2$ gets redistributed away from the perinuclear region. In order to measure how heterogeneous the protein distribution gets after cholesterol depletion we also measured the ratio standard deviation. The higher the standard deviation the higher is the discrepancy between $Ca_v1.2$ distribution between perinuclear and away from the perinuclear regions.

Statistical analysis

We performed Student's t-test comparing control and cholesterol depleted groups. Data was represented by mean values ± standard errors unless otherwise stated. Statistical differences were labeled with asterisks.

Supplemental references

1 Oakes, P. W., Beckham, Y., Stricker, J. & Gardel, M. L. Tension is required but not sufficient for focal adhesion maturation without a stress fiber template. *Journal of Cell Biology* **196**, 363-374, doi:10.1083/jcb.201107042 (2012).

2 Aratyn-Schaus, Y., Oakes, P. W., Stricker, J., Winter, S. P. & Gardel, M. L. Preparation of complaint matrices for quantifying cellular contraction. *Journal of visualized experiments : JoVE*, doi:10.3791/2173 (2010).

3 Maruthamuthu, V. & Gardel, M. L. Protrusive Activity Guides Changes in Cell-Cell Tension during Epithelial Cell Scattering. *Biophysical Journal* **107**, 555-563, doi:10.1016/j.bpj.2014.06.028 (2014).

4 Sabass, B., Gardel, M. L., Waterman, C. M. & Schwarz, U. S. High resolution traction force microscopy based on experimental and computational advances. *Biophysical Journal* **94**, 207-220, doi:10.1529/biophysj.107.113670 (2008).

5 Plotnikov, S. V., Sabass, B., Schwarz, U. S. & Waterman, C. M. High-Resolution Traction Force Microscopy. *Quantitative Imaging in Cell Biology* **123**, 367-394, doi:10.1016/b978-0-12-420138-5.00020-3 (2014).

6 Oakes, P. W., Banerjee, S., Marchetti, M. C. & Gardel, M. L. Geometry Regulates Traction Stresses in Adherent Cells. *Biophysical Journal* **107**, 825-833, doi:10.1016/j.bpj.2014.06.045 (2014).

7 Butler, J. P., Tolic-Norrelykke, I. M., Fabry, B. & Fredberg, J. J. Traction fields, moments, and strain energy that cells exert on their surroundings. *American Journal of Physiology-Cell Physiology* **282**, C595-C605 (2002).

8 Pontes, B. *et al.* Cell cytoskeleton and tether extraction. *Biophysical journal* **101**, 43-52, doi:10.1016/j.bpj.2011.05.044 (2011).

9 Pontes, B. *et al.* Membrane Elastic Properties and Cell Function. *Plos One* **8**, doi:10.1371/journal.pone.0067708 (2013).

10 Hissa, B. *et al.* Membrane cholesterol removal changes mechanical properties of cells and induces secretion of a specific pool of lysosomes. *PloS one* **8**, e82988-e82988 (2013).

11 Bauer, B., Davidson, M. & Orwar, O. Direct reconstitution of plasma membrane lipids and proteins in nanotube-vesicle networks. *Langmuir* **22**, 9329-9332, doi:10.1021/la060828k (2006).

12 Guatimosim, S., Guatimosim, C. & Song, L.-S. Imaging Calcium Sparks in Cardiac Myocytes. *Light Microscopy: Methods and Protocols* **689**, 205-214, doi:10.1007/978-1-60761-950-5_12 (2011).

13 Gomez, J. P., Potreau, D. & Raymond, G. Intracellular calcium transients from newborn rat cardiomyocytes in primary culture. *Cell Calcium* **15**, 265-275, doi:10.1016/0143-4160(94)90066-3 (1994).
